# Vitamin D Supplementation for Patients with Dysmenorrhoea: A Meta-Analysis with Trial Sequential Analysis of Randomised Controlled Trials

**DOI:** 10.3390/nu16071089

**Published:** 2024-04-08

**Authors:** Kan-Chu Lin, Kuan-Ju Huang, Ming-Nan Lin, Cheng-Yu Wang, Tou-Yuan Tsai

**Affiliations:** 1 Family Medicine Department, Dalin Tzu Chi Hospital, Buddhist Tzu Chi Medical Foundation, Chiayi 62247, Taiwan; fxvictoria1994@gmail.com (K.-C.L.); mingnan.lin@gmail.com (M.-N.L.); 2 Department of Obstetrics and Gynecology, National Taiwan University Hospital Yunlin Branch, Yunlin 640203, Taiwan; restroomer@icloud.com; 3 School of Medicine, Tzu Chi University, Hualien 97004, Taiwan; 4 Emergency Department, Dalin Tzu Chi Hospital, Buddhist Tzu Chi Medical Foundation, Chiayi 62247, Taiwan; 5 Institute of Epidemiology and Preventive Medicine, College of Public Health, National Taiwan University, Taipei 106319, Taiwan

**Keywords:** dysmenorrhoea, effectiveness, meta-analysis, pain, systematic review, trial sequential analysis, vitamin D supplementation

## Abstract

Vitamin D reduces prostaglandin levels and inflammation, making it a promising treatment option for dysmenorrhoea. However, its effects on pain intensity in different types of dysmenorrhoea remain unclear. We examined whether vitamin D supplementation decreases pain intensity in patients with dysmenorrhoea. The Cochrane Library, Embase, Google Scholar, Medline, and Scopus databases were searched from inception to 30 December 2023. Randomised controlled trials (RCTs) evaluating vitamin D supplementation effects on such patients were included. The primary and secondary outcomes were measured by the changes in pain intensity and rescue analgesic use, respectively. Pooled mean differences and rate ratios were calculated using a random-effect model; trial sequential analysis (TSA) was also performed. Overall, 11 studies involving 687 participants were included. Vitamin D supplementation significantly decreased pain intensity in patients with dysmenorrhoea compared with controls (pooled mean difference, −1.64; 95% confidence interval, −2.27 to −1.00; *p* < 0.001; CoE, moderate; *I*^2^ statistic, 79.43%) and indicated substantial heterogeneity among the included studies. TSA revealed that the current RCTs provide sufficient information. In subgroup analyses, vitamin D supplement reduced primary dysmenorrhoea pain but not secondary dysmenorrhoea pain. In conclusion, although substantial heterogeneity persists, vitamin D supplementation decreased pain intensity in patients with dysmenorrhea, especially in those with primary dysmenorrhoea.

## 1. Introduction

Dysmenorrhoea, characterised by painful menstrual periods or cramps, is a prevalent condition affecting 45–95% of female individuals, particularly adolescents and young women [1]. It is classified into primary dysmenorrhoea, where no underlying medical condition is present, and secondary dysmenorrhoea, which is associated with specific medical conditions [2]. Dysmenorrhoea represents a significant global health burden that requires attention [3]. Healthcare costs for patients with dysmenorrhoea are 2.2 times those for the general population [4]. Additionally, in some countries, the annual cost associated with dysmenorrhoea treatment is 25 million USD [5].

Nonsteroidal anti-inflammatory drugs (NSAIDs) are typically the first-line treatment for dysmenorrhoea. However, they can cause kidney injury, gastrointestinal bleeding, and are associated with high rates of hypersensitivity reactions [6,7]. Hormone therapy is another treatment option, but concerns exist regarding its long-term effects on cardiovascular health and cancer risk [7,8,9].

Evidence suggests that vitamin D supplementation contributes to pain relief [10,11]. Vitamin D inhibits pain-sensing signals at the dorsal root ganglion and reduces inflammation factors [12,13,14]. Vitamin D deficiency has been reported to induce or exacerbate dysmenorrhoea symptoms [14]. Notably, several randomised controlled trials (RCTs) have demonstrated that vitamin D supplementation reduces pain intensity in patients with dysmenorrhoea; thus, this may be an alternative treatment option for dysmenorrhoea [15,16,17,18,19,20,21,22]. However, other studies have reported limited effects [23,24,25]. Of note, the level of vitamin D supplementation, as well as dosage and frequency, varies between current studies.

A recent systematic review reported that vitamin D supplementation significantly reduced the pain level in patients with primary dysmenorrhoea [26]. However, its effects on pain intensity in different types of dysmenorrhoea remain unclear. Therefore, in this systematic review and meta-analysis of RCTs, we aimed to assess the effectiveness of vitamin D supplementation in alleviating dysmenorrhoea-related pain. Additionally, we conducted trial sequential analysis (TSA) to assess the statistical power and precision of the meta-analysis and identify the ‘true’ intervention effect.

## 2. Materials and Methods

### 2.1. Study Protocol

This systematic review was performed and reported according to the Preferred Reporting Items for Systematic Reviews and Meta-Analyses Statement 2020 [27]. The study protocol was approved by the Institutional Review Board of Dalin Tzu Chi Hospital, Buddhist Tzu Chi Medical Foundation, Taiwan (B11201016). The study was registered with PROSPERO under code CRD42023394841.

### 2.2. Search Strategy

The Cochrane Library, Embase, Google Scholar, Medline, and Scopus databases were systematically searched from inception to 30 December 2023, using the following keywords: ‘dysmenorrhea’, ‘vitamin D supplement’, and ‘pain intensity’. Medical Subject Headings and Emtree terms were combined using a Boolean search strategy without any limits applied. References from included articles were screened to identify potentially relevant studies. Table S1 presents the full search strategy.

Studies were included if they met the following criteria: (1) RCT, (2) involving women who had regular menstruation, (3) comparing vitamin D supplementation with placebo or other active treatments before and after their use, and (4) assessing pain severity using a validated tool. No restrictions were applied to language, sample size, or publication date. The exclusion criteria encompassed studies involving pregnant or menopausal participants, those lacking a placebo group, animal studies, non-RCTs, review articles, case reports, editorials, and letters.

### 2.3. Outcome Measures

The primary outcome of this study aimed to evaluate treatment response by measuring the change in pain intensity (also known as pain score reduction) using established tools, such as the numeric rating scale or visual analogue scale. Pain intensity difference (PID) is defined as the difference in pain scores between any observation time and the baseline. The secondary outcome focused on determining the ratio of patients using rescue analgesics.

### 2.4. Data Extraction and Assessment of Methodological Quality

Two reviewers (KCL and CYW) independently screened the titles and abstracts of all included articles, assessed the full text of the studies, and extracted the data. In cases of disagreements concerning the inclusion or exclusion of a study, a third reviewer (TYT) was consulted, and a consensus was reached. Data extraction was further reviewed and double-checked by the third reviewer. The extracted data included the first author’s name, publication year, study design, country of study, number of participants, age of participants, prescriptions of vitamin D dosage and frequency, pain intensity before and after vitamin D supplementation, and duration of follow-up in RCTs. When data were not readily available or lacked clarity, corresponding authors were contacted for clarification.

We assessed the risk of bias using the Cochrane Collaboration RoB-2 tool, which consisted of five domains evaluated as ‘low risk of bias’, ‘some concerns’, or ‘high risk of bias [28]’. A study was considered to have a ‘high risk of bias’ if at least one domain was evaluated to have a ‘high risk of bias’ or multiple domains were evaluated as having ‘some concerns’ substantially diminishing confidence in the results. Both reviewers deliberated on bias risk in every domain, and the third reviewer cross-checked the overall bias. If studies were identified as having a high risk of bias, meta-analyses were conducted after stratifying the studies based on their quality.

### 2.5. Data Collection, Processing, and Analysis

We calculated the pooled mean differences (MDs) of PID and the corresponding 95% confidence interval (CIs) for the primary outcome using a random-effect model owing to possible heterogeneity among articles. Mean changes in each group were computed by subtracting baseline means from final means if not explicitly provided. Standard deviations were computed using the equation in the Cochrane Handbook [29]. As the baseline and final pain scores were measured by the same person, we used an intra-individual correlation of 0.25, as previously reported [30]. Pooled rate ratios (RRs) with 95% CIs were calculated for the secondary outcomes. We added 0.5 to all cells of the 2 × 2 table to avoid zero cells. We evaluated inter-study heterogeneity using the *I*^2^ statistic and interpreted *I*^2^ values of 25%, 50%, and 75% as low, moderate, and high heterogeneity, respectively [31]. Meta-regressions were conducted to explore potential effect modifiers. Funnel plots were constructed and the Begg adjusted rank correlation test performed to assess small-study bias and potential publication bias [32,33]. We conducted a leave-one-out sensitivity analysis (performed by omitting one study at a time) to evaluate the influence of each study on the overall pooled estimate. Moreover, we performed other sensitivity analyses, including risk of bias, blind method, funding support, and placebo type. Statistical analyses were performed using STATA version 17.0 (StataCorp, College Station, TX, USA). All statistical tests were two-sided. Statistical significance was set at *p* < 0.05.

Further, we performed subgroup analyses based on the dysmenorrhoea type, serum 25-hydroxyvitamin D (25(OH)D) levels, frequency and dosage of vitamin D, and study country. Dysmenorrhoea was classified according to American College of Obstetricians and Gynecologists guidelines [2]. Primary dysmenorrhoea was defined as painful menstrual cramps occurring without underlying pelvic abnormalities or disorders. Pain intensity typically peaks at 24–48 h after the onset of menses and lasts up to 72 h [34,35]. Secondary dysmenorrhoea was defined as having painful menstruation caused by pelvic pathology or a medical condition. Vitamin D deficiency was defined as having a serum hydroxyvitamin D (25(OH)D) level below 30 ng/mL (75 nmol/L) according to Endocrine Society Clinical Practice guidelines [36].

### 2.6. Trial Sequential Analysis

To decrease the risk of type I errors, we conducted TSA using software version 0.9 beta (Copenhagen Trial Unit, Centre for Clinical Intervention Research, Copenhagen, Denmark). We selected the DerSimonian and Laird method to assess random effects. For hypothesis testing, we applied O’Brien–Fleming monitoring boundaries to avoid type I errors. The computed required information size was based on an α-value of 0.05 (two-sided) and β-value of 0.20 (power 80%). The mean difference effect was estimated from the random-effect model, incorporating the estimated variance and heterogeneity observed in the included trials [37]. Statistical significance was set as the cumulative Z-curve crossing the TSA boundaries. Specifically, if the Z-curve crossed the O’Brien–Fleming boundaries before reaching the estimated required information size (RIS) or if the Z-curve exceeded 1.96 when the accumulated size exceeded the RIS, these outcomes were considered true positives. Conversely, if the Z-curve entered the futility area, it was deemed a true negative. A total sample size that failed to reach the RIS was classified as underpowered.

### 2.7. Grading of the Certainty of Evidence (CoE)

KCL and CYW assessed the CoE using the Grading of Recommendations Assessment, Development and Evaluation (GRADE) methodology to assess the quality of evidence with individual endpoints. After step-by-step evaluation, CoE was classified as high, moderate, low, or very low [38].

## 3. Results

**Figure 1:**
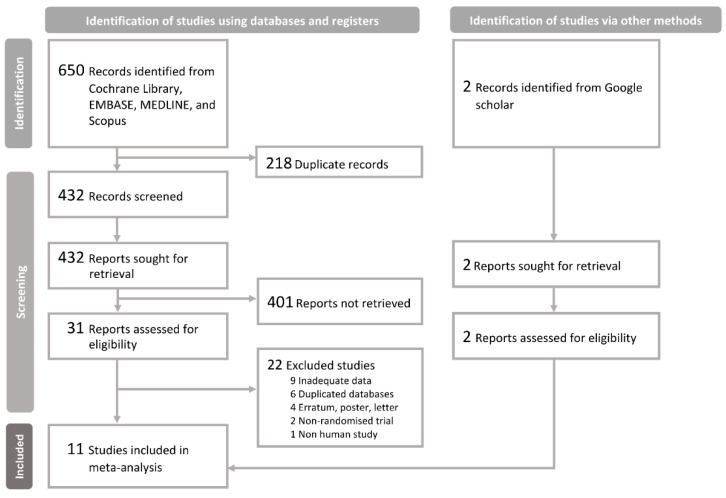
Literature search and article selection.

Figure 1 shows the literature search and selection process. After the initial screening of article titles and abstracts, 31 studies were identified as potentially relevant and subjected to full review. Of them, 22 studies were excluded.

We excluded the study by Zangene et al. as it utilised the same database (patients, study period, hospital, and results) as that of Ataee et al. [16,39]. Additionally, the study conducted by Moini et al. was excluded because pain intensity was analysed as ordinal categories [40]. Despite our attempts to obtain details of pain scores by contacting the first and corresponding authors via email, as of 5 March 2024, we had received no response. Ultimately, 11 studies with a total of 687 participants were included in the meta-analysis [15,16,17,18,19,20,21,22,23,24,25].

The characteristics of the included studies are presented in Table 1, with the quality of evidence assessed using the Cochrane Collaboration tool (Figure S1). Among the 11 studies, 3 were not blinded and had unclear definitions of the placebo group [18,19,20]. Of these studies, seven were conducted in Iran, and one each in Italy, Saudi Arabia, Turkey, and the USA. Eight studies enrolled patients with primary dysmenorrhoea, while three included those with secondary dysmenorrhoea. Furthermore, only three studies enrolled patients with vitamin D deficiency, all of whom had primary dysmenorrhoea [16,21,22]. Considerable variability was noted in the dosage, frequency, and duration of vitamin D supplementation across the included studies.

### 3.1. Primary and Secondary Outcomes

**Figure 2:**
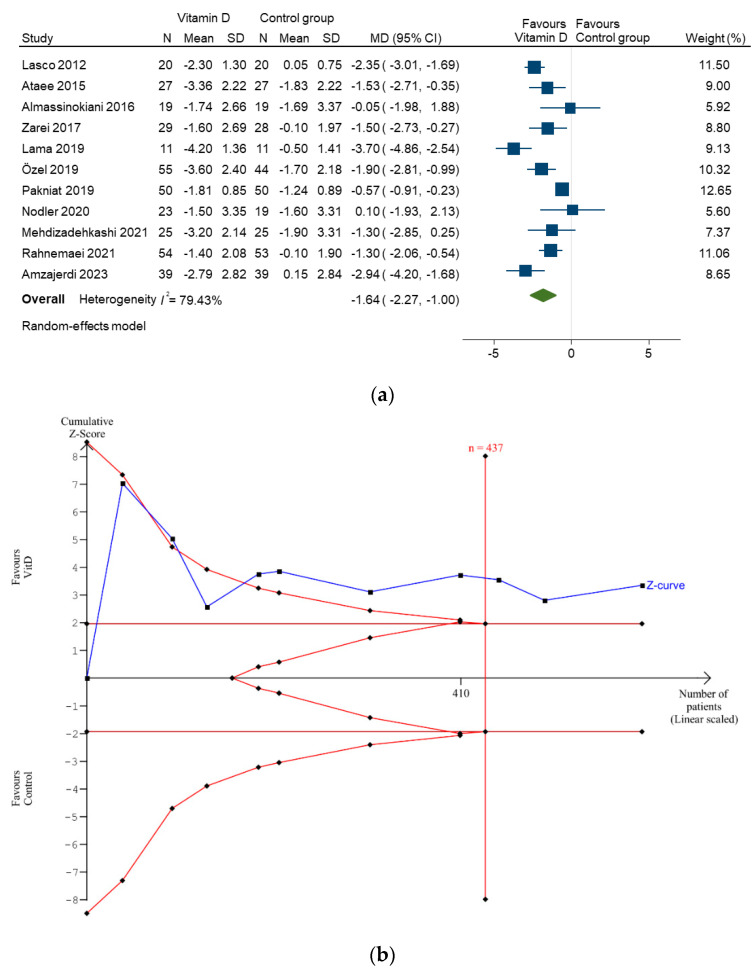
(**a**) Forest plot and (**b**) trial sequential analysis of pain intensity difference in patients with dysmenorrhoea supplemented with vitamin D. CI, confidence interval; MD, mean difference; N, number; SD, standard deviation [15,16,17,18,19,20,21,22,23,24,25].

In the primary meta-analysis of 687 patients in the 11 studies, vitamin D supplementation significantly decreased pain intensity in patients with dysmenorrhoea (pooled MD, −1.64; 95% CI, −2.27 to −1.00; *p* < 0.001; Figure 2a). The *I*^2^ statistic was 79.43% (*p* < 0.001), indicating substantial heterogeneity among the included studies. TSA results showed that a cumulative Z-curve across the O’Brien–Fleming boundaries after the RIS (437 participants for required power) was reached (Figure 2b). A true-positive result indicated that the cumulative power from the available literature supports a ‘true’ treatment response to vitamin D supplementation. No evidence of publication bias was found using Begg’s test (*p* = 0.28). The funnel plot is shown in Figure S2.

**Figure 3:**
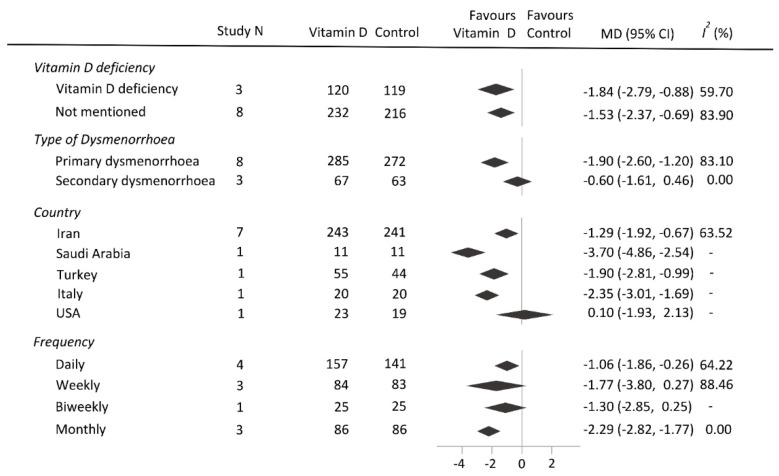
Subgroup analysis of pain intensity difference. CI, confidence interval; MD, mean difference; N, number.

In the subgroup analyses, vitamin D supplementation significantly decreased pain intensity of patients, with vitamin D deficiency compared with the corresponding of those in the control group (pooled MD, −1.84; 95% CI, −2.79 to −0.88; *p* < 0.001; Figure 3). In patients with primary dysmenorrhoea, vitamin D supplementation also decreased pain intensity (pooled MD, −1.90; 95% CI, −2.60 to −1.20; *p* < 0.001). Furthermore, both daily (pooled MD, −1.06; 95% CI, −1.86 to −0.26, *p* < 0.001) and monthly (pooled MD, −2.29; 95% CI, −2.82 to −1.77, *p* < 0.001) administration of vitamin D supplementation proved to be effective.

The results of the leave-one-out sensitivity analysis demonstrated the robustness of the pooled results. For instance, upon exclusion of the study conducted by Özel et al. in 2019 [19], the pooled estimate changed minimally from −1.64 to −1.60 (95% CI, −2.31 to −0.89; *p* < 0.001; Figure S3). Sensitivity analyses, including ‘meta-analysis with a fixed-effect model’, ‘studies with low risk of bias’, ‘double-blinded studies’, ‘non-funded studies’, and ‘studies with a definitive placebo’, consistently showed robustness compared to the primary outcome (Table S2).

Regarding the secondary outcome, only two of the eleven included studies compared the number of events involving the use of rescue analgesics between the groups with and without vitamin D supplementation. Both studies focused on patients with primary dysmenorrhoea. In the study conducted by Lasco et al. in 2012, none of the participants in the vitamin D supplement group used rescue NSAIDs during the 2-month study period, while 40% of women in the placebo group took NSAIDs at least once [15]. In the study by Özel et al., patients receiving vitamin D supplements required significantly fewer NSAIDs compared with those without [19]. The meta-analysis indicated a trend towards decreased use of rescue analgesics among patients with dysmenorrhoea receiving vitamin D supplementation; however, this result did not reach statistical significance (pooled rate ratio, 0.26; 95% CI, 0.05–1.33; *p* = 0.10; Figure S4). The wide confidence interval of the pooled estimate suggests insufficient power to conclusively support this finding, highlighting the need for further studies with larger samples.

### 3.2. GRADE Assessment

The GRADE assessment is shown in Table 2. For the primary outcome, we downgraded the overall CoE in the inconsistency domain because high heterogeneity was found in the meta-analysis. Overall, the CoE was moderate, supporting the conclusion that vitamin D supplementation decreased pain intensity in patients with dysmenorrhoea compared with the control group. For the secondary outcome, we downgraded the domain of ‘risk of bias’ and ‘imprecision’, resulting in a very low CoE for rescue analgesic use.

## 4. Discussion

### 4.1. Main Findings

There are conflicting reports on the benefits of vitamin D supplementation in alleviating pain intensity of dysmenorrhoea. A previous systematic review revealed that vitamin D supplementation can relieve dysmenorrhoea; however, this review did not provide statistical data to support such an effect [13]. Meanwhile, another meta-analysis reported that vitamin D supplementation did not affect dysmenorrhoea; however, this study primarily focused on patients with endometriosis [41]. A recent systematic review showed that vitamin D supplementation substantially reduced the pain levels in patients with primary dysmenorrhoea, but the study did not explore the effect of vitamin D supplementation on those with secondary dysmenorrhoea [26]. To our knowledge, this is the first meta-analysis of RCTs assessing the effectiveness of vitamin D supplementation in patients with different types of dysmenorrhoea. Evidence obtained from 11 RCTs was evaluated and analysed using TSA. In the pooled analyses, we found that vitamin D supplementation significantly decreased the pain intensity of dysmenorrhoea, and the cumulative power supports a ‘true’ treatment response. Subgroup analyses revealed that vitamin D supplementation was effective in reducing pain intensity in patients with vitamin D deficiency and those with primary dysmenorrhoea. Overall, our study provides evidence on the effectiveness of vitamin D supplementation for patients with dysmenorrhoea.

### 4.2. Interpretation

A recent systematic review demonstrated that vitamin D treatment reduced the severity of pain in women with primary dysmenorrhoea, which aligns with the findings of our study [26]. However, our study revealed differing effects between primary and secondary dysmenorrhoea. In addition to utilising subjective pain scores as the primary outcome, we also assessed the outcome of rescue analgesic use in the included studies, offering an alternative perspective on the relationship between vitamin D use and dysmenorrhoea. Furthermore, we performed trial sequential analysis to adjust for the inflated type 1 error rate resulting from multiple meta-analyses on the same topics during the accumulation of evidence and to estimate the statistical power and required sample size, which was not undertaken in the previous systematic review. These findings contribute valuable insights for clinical decision-making.

While a previous study proposed an association between underlying vitamin D concentration and various pain-related conditions [42], the specific effect of vitamin D on dysmenorrhoea remains unclear. Vitamin D receptors are found in the ovaries, uterus, placenta, and pituitary gland [42,43], and vitamin D suppresses the expression of inflammation-induced markers and contractile-associated factors in uterine myometrial smooth-muscle cells by interacting with these receptors [44]. Notably, a decrease in serum vitamin D (25(OH)D) levels was observed in the luteal phase of the menstrual cycle [45], which may stimulate an increase in inflammatory cytokines and prostaglandins, exacerbating the pain intensity of dysmenorrhoea. Overall, through these mechanisms, vitamin D supplementation offers positive benefits in alleviating the pain severity of dysmenorrhoea. In our study, subgroup analyses showed that vitamin D supplementation effectively alleviates dysmenorrhoea in patients with vitamin D deficiency. Although the other included studies did not specifically target patients with vitamin D deficiency, the baseline serum vitamin D (25(OH)D) levels were relatively low [15,18,24,25].

Vitamin D was found to decrease invasion and proliferation of endometriotic lesions, and vitamin D deficiency is a known risk factor of endometriosis [41,46]. In the subgroup analysis of our meta-analysis, all three studies conducted with secondary dysmenorrhoea including patients with endometriosis, vitamin D supplementation was effective in relieving the pain of secondary dysmenorrhoea, albeit this was not statistically significant [23,24,25]. One of the three studies reported a neutral effect from vitamin D supplementation. It included patients with endometriosis following laparoscopic surgery [23]. Moreover, the severity of endometriosis presented considerable variation across these included studies. Further investigations are imperative to comprehend the effectiveness of vitamin D supplementation on patients with secondary dysmenorrhoea.

The differential impact of vitamin D supplementation, stratified by different countries, may introduce potential confounding factors. Among the seven studies conducted in Iran, three included patients with definite vitamin D deficiency [16,21,22], whereas the remaining studies included those with relatively low serum vitamin D (25(OH)D) level [16,21,22,25]. The prevalence of vitamin D deficiency is as high as 64% in Iranian women [47]. In our meta-analysis, the other included studies, which enrolled Iranian women with unknown serum vitamin D (25(OH)D) levels, may have substantially included those with relatively low vitamin D levels.

Although the current guideline recommends a weekly vitamin D dose of 50,000 IU for 8 weeks for treating vitamin D deficiency, the appropriate frequency for treating dysmenorrhoea remains controversial [36]. In our meta-analysis, the frequency of vitamin supplement usage varied among the included studies (Table 1). The study conducted by Zarei et al. administered vitamin D supplements to patients once daily from day 15 of the menstrual cycle until the end of dysmenorrhoea [17]. In contrast, the study by Rahnemaei et al. opted for a weekly treatment regimen [21]. Two of the included studies provided treatment once daily for 5 days during the menstrual cycle, while three studies administered a single high dose of vitamin D [15,16,19,20,22]. These varying frequencies of vitamin D supplement usage all appear to be effective in reducing dysmenorrhoea-associated pain. Although the studies conducted by Lasco et al., Ataee et al., and Amzajerdi et al. administered doses exceeding the recommended 50,000 IU per week (equivalent to 200,000 IU per menstruation cycle), the patients in the studies of Ataee et a. and Amzajerdi et al. were all identified as having vitamin D deficiency [15,16,22]. Furthermore, post-treatment serum vitamin D (25(OH)D) levels did not surpass the 150 ng/mL limit recommended by guidelines in these studies. Moreover, none of the patients reported symptoms of vitamin D toxicity after treatment, such as neurological, gastrointestinal, or renal symptoms or bone pain [48].

### 4.3. Strengths and Limitations

The main strengths of our meta-analysis lie in the inclusion of results from RCTs, a study design known for its ability to control potential confounding and unmeasured confounders. Additionally, the implementation of TSA ensures that the collective evidence possesses adequate statistical power.

However, this review has some limitations. First, the limited number of available RCTs in this field restricted our ability to fully address potential study biases. Moreover, only eight of the eleven RCTs were double-blinded. Second, the results exhibited considerable multifactorial heterogeneity, including variations in the type of dysmenorrhoea, dosage and frequency of supplementation, follow-up duration, and differences in geographic location and racial demographics. Third, while subgroup analyses and meta-regression (R^2^ = 0%, *p* = 0.66) indicated that vitamin D supplementation reduces dysmenorrhoea pain independently of the baseline vitamin D deficiency status, it remains uncertain whether the effectiveness of vitamin D supplements in dysmenorrhea treatment is due to correcting vitamin D deficiency or a pharmacological effect. Fourth, most estimates in the subgroup analysis stratified by geographic location were derived from a single study, warranting cautious interpretation of the results. Fifth, although we made efforts to include only high-quality studies, some studies had a high risk of bias, which may have affected our overall conclusions. Nevertheless, the meta-regression revealed that the risk of bias did not represent an effect modifier (adjusted R^2^ = 0.00%, *p* = 0.68). Additionally, sensitivity analyses excluding studies with unblinded methods and funded or unclear placebo designs showed similar results to our primary analysis, suggesting the robustness of our findings.

## 5. Conclusions

This systematic review and meta-analysis showed that vitamin D supplementation significantly decreases pain intensity and rescue analgesic use in patients with dysmenorrhoea. The subgroup analyses revealed that vitamin D supplementation is effective for pain relief in patients with vitamin D deficiency and those with primary dysmenorrhoea. Owing to the inter-study heterogeneity, these results should be interpreted cautiously, and further large-scale high-quality studies are necessary to confirm our findings.

## Figures and Tables

**Table 1:** Summary of the included studies.

Study	Blinding	Country	Sample Size	Age Range (Years)	Type of Dysmenorrhea	Vit. D Deficiency	Baseline Serum 25(OH)D Level (ng/mL) ^†^	Vit. D Frequency	Vit. D (IU) per Dose	Vit. D (IU) per Cycle	Follow-Up Duration	Outcome Measurement
Lasco et al., 2012 [15]	Double-blinded	Italy	40	18–40	Primary	NA ^±^	Vit. D: 30.0 ± 7.6 Control: 27.3 ± 7.5	Once per month	300,000	300,000	2 months	VAS
Ataee et al., 2015 [16]	Double-blinded	Iran	54	18–30	Primary	Included ^#^	Vit. D: 7.3 ± 3.6 Control: 6.3 ± 2.8	Once per month	300,000	300,000	3 months	VAS
Almassinokiani et al., 2016 [23]	Double-blinded	Iran	38	15–40	Secondary	NA	NA	Once per week	50,000	200,000	16 weeks	VAS
Zarei et al., 2017 [17]	Double-blinded	Iran	57	18–32	Primary	NA	NA	Once per day ^§^	5000	65,000	3 months	VAS
Lama et al., 2019 [18]	Non-blinded	Saudi Arabia	22	13–40	Primary	NA ^±^	Vit. D: 30.1 ± 13.4, Control: 33.9 ± 11.9	Once per week	50,000	200,000	8 weeks	VAS
Özel et al., 2019 [19]	Non-blinded	Turkey	99	16–35	Primary	NA	NA	Once per day for 5 days	667	3335	2 months	VAS
Pakniat et al., 2019 [20]	Single-blinded	Iran	100	18–25	Primary	NA	NA	Twice per day for 5 days	12,500 *	125,000	2 months	VAS
Nodler et al., 2020 [24]	Double-blinded	USA	42	12–25	Secondary	NA	Vit. D: 33.8 ± 11.9 Control: 31.2 ± 12.0	Once per day	2000	56,000	6 months	VAS
Mehdizadehkashi et al., 2021 [25]	Double-blinded	Iran	50	18–40	Secondary	NA	Vit. D: 24.7 ± 7.6 Control: 25.4 ± 10.0	Biweekly	50,000	100,000	12 weeks	NA
Rahnemaei et al., 2021 [21]	Double-blinded	Iran	107	18–32	Primary	Included	Vit. D: 20.0 ± 6.0 Control: 19.5 ± 5.5	Once per week	50,000	200,000	8 weeks	NRS
Amzajerdi et al., 2023 [22]	Double-blinded	Iran	78	18–25	Primary	Included	Vit. D: 5.1 ± 3.3 Control: 6.6 ± 5.6	Once per month	300,000	300,000	2 months	VAS

Abbreviations: 25(OH)D, 25-hydroxyvitamin D; NA, not available; NRS, numeric rating scale; VAS, visual analogue scale. * Vitamin D group: 1000 mg vitamin D tablet (D-VIGEL; Dana Co., New York, NY, USA) per day, which is equal to 25,000 IU per day. ^†^ Data are presented as means ± SDs. ^±^ Despite the aims of Lasco et al. [15] and Lama et al. [18] to include participants with vitamin D deficiency, their baseline 25(OH)D levels did not meet the Endocrine Society’s criteria (<30 ng/mL). Therefore, these studies were not classified as focusing on vitamin D deficiency. ^#^ The study by Ataee et al. [16] did not specifically target patients with vitamin D deficiency, but their participants had very low baseline 25(OH)D levels. Therefore, we classified it as a study on vitamin D deficiency. ^§^ From day 15 of menstrual cycle to the end of dysmenorrhea.

**Table 2:** Certainty of evidence evaluated using the Grading of Recommendations Assessment, Development and Evaluation (GRADE) methodology.

No. of Trials (No. of Patients)	Risk of Bias	Inconsistency	Indirectness	Imprecision	Publication Bias	Effect (95% CI)	Overall Quality of Evidence
Pain intensity reduction							
11 (687)	No concerns	Downgraded *I*^2^ = 79.43%	Not downgraded	Not downgraded	Not downgraded Begg’s test = 0.28	MD = −1.64 (−2.27 to −1.00)	⊕⊕⊕⊖ MODERATE
Rescue use of analgesics							
2 (139)	Downgraded	Not downgraded *I*^2^ = 45.28 %	Not downgraded	Downgraded	NA	RR = 0.26 (0.05–1.33)	⊕⊖⊖⊖ VERY LOW

CI, confidence interval; MD, mean difference; NA, not available; RR, rate ratio. GRADE working group evidence. High certainty: We are very confident that the true effect lies close to that of the estimate of the effect. Moderate certainty: We are moderately confident in the effect estimate. The true effect is likely to be close to the estimate of the effect, but there is a possibility that it is substantially different. Low certainty: Our confidence in the effect estimate is limited. The true effect may be substantially different from the estimate of the effect. Very low certainty: We have very little confidence in the effect estimate. The true effect is likely to be substantially different from the estimate of the effect.

## Data Availability

The datasets used and analyzed during the current study are available from the corresponding author upon reasonable request.

## Supplementary Materials

The following supporting information can be downloaded at: https://www.mdpi.com/article/10.3390/nu16071089/s1, Table S1. Search Strategy, Table S2. Sensitivity analyses for the primary outcome, Figure S1. Quality assessment of the included studies using the RoB-2 tool, Figure S2. Funnel plot, Figure S3. Leave-one-out analysis, Figure S4. The use of rescue analgesics among patients with dysmenorrhoea.
