# Supplementary material for: Vitamin D Supplementation for Patients with Dysmenorrhoea: A Meta-Analysis with Trial Sequential Analysis of Randomised Controlled Trials
Source: Nutrients. 2024 Apr 8;16(7):1089. doi: 10.3390/nu16071089 (PMC11013696; doi:10.3390/nu16071089)
Supplement: Supplementary file 1 [file nutrients-16-01089-s001.zip › nutrients-2925012-supplementary.pdf]

Supplementary Materials

Vitamin D Supplementation for Patients with Dysmenorrhoea: A Meta-analysis with Trial Sequential Analysis of Randomized Control Trials

Supplementary Table S1. Search Strategy 2

Supplementary Table S2. Sensitivity analyses for the primary outcome 11

Supplementary Figure S1. Quality assessment of the included studies using the RoB-2 tool 12

Supplementary Figure S2. Funnel plot 13

Supplementary Figure S3. Leave-one-out analysis 14

Supplementary Figure S4. The use of rescue analgesics among patients with dysmenorrhoea 15

Supplementary Table S1. Search Strategy

search date:2023/6/25

Database	#	Search Syntax	Result
**Embase.com (Elsevier)**	1	"dysmenorrhea"/exp OR "menstruation disorder"/de OR "pelvic pain"/de OR "endometriosis"/de	
	2	(dysmenorrhea* OR dys-menorrhea* OR dysmenorrhoea* OR dys-menorrhoea* OR cramping OR endometrios* OR endometrioma* OR "adenomyos* externa" OR "e. externa" OR ((catamenial OR menstrua* OR menses OR premenstrua* OR period* OR pelvic OR pelvis) NEAR/4 (pain* OR ache* OR cramp* OR distress*))):ti,ab,kw	
	3	"vitamin D"/exp	
	4	((vitamin* NEAR/4 D*) OR vitaminD* OR "vit D*" OR "vita D*" OR cholecalciferol* OR ergocalciferol*):ti,ab,kw	
	5	(epivitamin* OR difvitamin* OR didehydrovitamin* OR dihydroxyvitamin* OR hydroxyvitamin* OR "25(OH)D" OR norvitamin* OR oxavitamin* OR oleovitamin* OR trihydroxyvitamin* OR epicholecalciferol* OR dihydroxycholecalciferol* OR didehydrocholecalciferol* OR hydroxycholecalciferol* OR norcholecalciferol* OR trihydroxycholecalciferol* OR epiergocalciferol* OR dihydroxyergocalciferol* OR hydroxyergocalciferol* OR trihydroxyergocalciferol* OR colecalciferol OR epicolecalciferol* OR dihydroxycolecalciferol* OR hydroxycolecalciferol* OR norcolecalciferol* OR oxacolecalciferol OR trihydroxycolecalciferol* OR dihydrotachysterol*):ti,ab,kw	
	6	("1 [1alpha, 3beta dihydroxy 9, 10 secochola 5, 7, 10 (19) trien 24 oyl] piperidine" OR "1 fluoro 26, 27 dihomo 9, 10 secocholesta 5, 7, 10 (19), 16, 23 pentaene 3, 25 diol" OR "1, 3, 24 trihydroxy 9, 10 secocholesta 5, 7, 10 (19), 22 tetraene 25 carboxylic acid isopropyl ester" OR "19 nor 1, 25 dihydroxycalciferol" OR "19 nor 9, 10 seco 14beta cholesta 5, 7 dien 23 yne 1alpha, 3beta, 25 triol" OR "19 nor 9, 10 secoergosta 5, 7, 22 triene 1alpha, 3beta, 25 triol" OR "19 norcolecalciferol" OR "1alpha hydroxycalciferol" OR "1alpha, 25 dihydroxycalciferol" OR "1alpha-hydroxycalciferol" OR "1-alpha-hydroxycalciferol" OR "2 [(1, 3 dihydroxy 9, 10 secopregna 5, 7, 10 (19), 16 tetraen 20 yl) oxy] n (2, 2, 3, 3, 3 pentafluoropropyl) acetamide" OR "2 [(1alpha, 3beta dihydroxy 9, 10 secopregna 5, 7, 10 (19), 16 tetraen 20 yl) oxy] n (2, 2, 3, 3, 3 pentafluoropropyl) acetamide" OR "2 [1 [7 [2 (3, 5 dihydroxy 2 methylenecyclohexylidene) ethylidene] 3a, 4, 5, 6, 7, 7a hexahydro 3a methyl 1h inden 3 yl] ethoxy] n (2, 2, 3, 3, 3 pentafluoropropyl) acetamide" OR "20 [3 (1 hydroxy 1 methylethyl) benzyloxymethyl] 9, 10 secopregna 5, 7, 10 (19) triene 1alpha, 3beta diol" OR "20 [3 (2 hydroxypropan 2 yl) benzyloxymethyl] 9, 10 secopregna 5, 7, 10 (19) triene 1alpha, 3beta diol" OR "20 epi 22 ethoxy 24a,26a,27a trihomo 9,10 secocholesta 5,7,10(19) trien 23 yne 1alpha,3beta,25 triol" OR "20 epicalcitriol" OR "22 oxacalcitriol" OR "22, 23 dihydrocalciferol" OR "22,23 didehydrocalcitriol" OR "24 (2 methylpropane 2 sulfonyl) 9, 10 secochola 5, 7, 10 (19), 16, 23 pentaene 1, 3 diol" OR "24 cyclopropyl 9, 10 secochola 5, 7, 10 (19), 22 tetraene 1alpha, 3beta, 24 triol" OR "24 hydroxycalcidiol" OR "24 methyl 9, 10 secocholesta 5, 7, 10 (19), 22 tetraen 3 ol" OR "24, 25 dihydroxycalciferol" OR "24, 26, 27 trihomo 9, 10 secocholesta 5, 7, 10 (19), 22, 24 pentaene 1alpha, 3beta, 25 triol" OR "25 hydroxycalciferol" OR "26 hydroxycalcidiol" OR "26, 27 cyclo 9, 10 seco 5, 7, 10 (19), 22 cholestatetraene 1, 3, 24 triol" OR "2beta (3 hydroxypropoxy) 9, 10 secocholesta 5, 7, 10 (19) triene 1alpha, 3beta, 25 triol" OR "3 [2 [7a methyl 1 (6 methylheptan 2 yl) 2, 3, 3a, 5, 6, 7 hexahydro 1h inden 4 ylidene] ethylidene] 4 methylidenecyclohexan 1 ol" OR "4 [2 [1 (butan 2 yl) 7a methyloctahydro 4h inden 4 ylidene] ethylidene] 2 methylidenecyclohexane 1, 3 diol" OR "4 methylidene 5 [2 [7a methyl 1 [6 methylheptan 2 yl] 2, 3, 3a, 5, 6, 7 hexahydro 1h inden 4 ylidene] ethylidene] cyclohexane 1, 3 diol" OR "5 [2 [1 (5 hydroxy 1, 5 dimethylhexyl) 7a methyl 2, 3, 3a, 5, 6, 7 hexahydro 1h inden 4 ylidene] ethylidene] 4 methylenecyclohexane 1, 3 diol" OR "5 [2 [1 [5 cyclopropyl 5 hydroxypent 3 en 2 yl] 7a methyl 2, 3, 3a, 5, 6, 7 hexahydro 1h inden 4 ylidene] ethylidene] 4 methylidenecyclohexane 1, 3 diol" OR "5 [2 [1 [5, 6 dimethylhept 3 en 2 yl] 7a methyl 2, 3, 3a, 5, 6, 7 hexahydro 1h inden 4 ylidene] ethylidene] 4 methylidenecyclohexane 1, 3 diol" OR "5 [2 [1 [6 hydroxy 5, 6 dimethylhept 3 en 2 yl] 7a methyl 2, 3, 3a, 5, 6, 7 hexahydro 1h inden 4 ylidene] ethylidene] cyclohexane 1, 3 diol" OR "5 [2 [1 [6 hydroxy 6 methylheptan 2 yl] 7a methyl 2, 3, 3a, 5, 6, 7 hexahydro 1h inden 4 ylidene] ethylidene] 4 methylidenecyclohexane 1, 3 diol" OR "5 [2 [7a methyl 1 [6 methylheptan 2 yl] 2, 3, 3a, 5, 6, 7 hexahydro 1h inden 4 ylidene] ethylidene] 4 methylidenecyclohexane 1, 3 diol" OR "6 [4 [2 [5 hydroxy 2 methylidenecyclohexylidene] ethylidene] 7a methyl 2, 3, 3a, 5, 6, 7 hexahydro 1h inden 1 yl] 2 methyl 2, 3 heptanediol" OR "9, 10 seco 5, 7, 10 (19) cholestatrien 1alpha, 3beta diol" OR "9, 10 seco 5, 7, 10 (19) cholestatrien 3 ol" OR "9, 10 seco 5, 7, 10 (19) ergostatrien 3beta ol" OR "9, 10 seco 5, 7, 10 (19), 22 ergostatetraen 3beta ol" OR "9, 10 seco 5, 7, 10 (19), 22 ergostatetraene 1, 3 diol" OR "9, 10 seco 5, 7, 22 ergostatrien 3beta ol" OR "9, 10 secocholesta 5, 7, 10 (19) trien 3 ol" OR "9, 10 secocholesta 5, 7, 10 (19) triene 1alpha, 3beta diol" OR "9, 10 secocholesta 5, 7, 10 (19) triene 1alpha, 3beta, 25 triol" OR "9, 10 secocholesta 5, 7, 10 (19) triene 3beta, 24, 25 triol" OR "9, 10 secocholesta 5, 7, 10 (19) triene 3beta, 25 diol" OR "9, 10 secoergosta 5, 7, 10 (19) trien 3beta ol" OR "9, 10 secoergosta 5, 7, 10 (19), 22 tetraen 3 ol 19, 22 tetraen 3 ol" OR "9, 10 secoergosta 5, 7, 10, 22 tetraene 1, 3 diol" OR "9, 10 secoergosta 5, 7, 22 trien 3beta ol" OR "9,10 secocholesta 5,7,10(19) trien 23 yne 1,3,25 triol" OR "9,10 secocholesta 5,7,10(19) trien 23 yne 3,25 diol" OR	

	"9,10 secocholesta 5,7,10(19),16 tetraen 23 yne 1,3,25 triol" OR "9,10 secocholesta 5,7,10(19),22 tetraene 1,3,25,26 tetrol" OR "a.t.10" OR "abt 358" OR "abt358" OR "activated 7 dehydrocholesterol" OR "adrovance" OR "afj d2" OR "alcovit d2" OR "aldevit" OR "alfacalcidol" OR "alfarol" OR "alpha calcidiol" OR "alpha calcidol" OR "alpha d3" OR "alphacalcidol" OR "antitanil" OR "antitetanin" OR "antitetanine" OR "arachitol" OR "asord" OR "at10" OR "atecen" OR "atocalcitol" OR "baby d" OR "becocalcidiol" OR "bentavit" OR "betamethasone dipropionate plus calcipotriene hydrate" OR "betamethasone dipropionate plus calcipotriene" OR "betamethasone dipropionate/calcipotriene hydrate" OR "betamethasone dipropionate/calcipotriene" OR "bocatriol" OR "bonalfa" OR "bonealpha" OR "bonesil d flas" OR "bonesyl" OR "bonky" OR "bxl 628" OR "bxl628" OR "cabone" OR "cacit d3" OR "cal d or" OR "cal d vita" OR "calcamin" OR "calcamine" OR "calceos" OR "calci chew d3 flex" OR "calci chew d3" OR "calcial d" OR "calcichew d3 extra" OR "calcichew d3 forte" OR "calcichew d3 opti" OR "calcichew d3" OR "calcidol" OR "calcifediol" OR "calciferol derivative" OR "calciferol" OR "calciferovit" OR "calcigran forte flex" OR "calcigran forte" OR "calcigran" OR "calcijex" OR "calcimagon d3 uno" OR "calcimagon d3" OR "calcimagon extra d3" OR "calcinosefaktor" OR "calcio d" OR "calciol" OR "calcioral d3" OR "calcipotriene hydrate plus betamethasone dipropionate" OR "calcipotriene hydrate" OR "calcipotriene hydrate/betamethasone dipropionate" OR "calcipotriene plus betamethasone dipropionate" OR "calcipotriene" OR "calcipotriene/betamethasone dipropionate" OR "calcipotriol" OR "calcitetrol" OR "calcitriol" OR "calcium d" OR "calcium wyeth" OR "calcivit d forte" OR "calcivit d" OR "caldefix" OR "calderol" OR "caldevita" OR "cal-d-or" OR "cal-d-vita" OR "calisvit" OR "calperos d3" OR "caltrate d" OR "caraben sc" OR "cb 1093" OR "cb1093" OR "chemovit d" OR "chocola d" OR "cicarol" OR "citrihexal" OR "citrokalciumd" OR "colextra-d3" OR "condol" OR "curatoderm" OR "d arthrin" OR "d arthrine" OR "d crivit" OR "d mulsin" OR "d tracetten" OR "d vatine" OR "d vital" OR "d2 vita" OR "d3 vicotrat" OR "dagravit d calcium" OR "daivobet" OR "daivonex" OR "dalonev" OR "davitamon d" OR "davitan" OR "davitin" OR "davonex" OR "ddrops" OR "decaps" OR "decostriol" OR "dediol" OR "dedrogyl" OR "dee osterol" OR "dee ron" OR "deeosterol" OR "deeron" OR "dekristol" OR "delakmin" OR "delta monovit" OR "deltabios" OR "deltalin" OR "deltaline" OR "deltamonovit" OR "deltar" OR "deltasterolo" OR "deltavit" OR "deltius" OR "deradion" OR "deradione" OR "deratol" OR "dergosten" OR "desunin" OR "desyn" OR "desyne" OR "detalup" OR "detamine" OR "deterapion" OR "deterapione" OR "devaron" OR "devitan" OR "devitil" OR "devitol" OR "dht intensol" OR "di actol" OR "di drol" OR "diactol" OR "dibase (drug)" OR "dibiovit" OR "dichistrolum" OR "dichysterol" OR "dichystrol" OR "didrogyl" OR "didrol" OR "didue vita" OR "diergin" OR "diergine" OR "diferol" OR "difilina" OR "difix" OR "dihydral" OR "dihydrotachysterin" OR "dihydrotachysterine" OR "dihydrotachysterol 2" OR "dihydrotachysterol 3" OR "dikystrol" OR "dilavit" OR "diserinal" OR "disir" OR "disnal" OR "disterina" OR "disterine" OR "ditelos" OR "divit urto" OR "divitina" OR "divitine" OR "diviturto" OR "divonex" OR "dn 101" OR "dn101" OR "dohyfral d" OR "dovobet" OR "dovonex" OR "doxercalciferol" OR "drisdol" OR "dumovit d" OR "duphafral" OR "dupharinterfran" OR "d-vital forte" OR "d-vital" OR "dydrogil" OR "dygratyl" OR "dz idrosol" OR "eb 1089" OR "eb1089" OR "ecalcidene" OR "ecatrol f" OR "ecatrol" OR "ed 71" OR "ed71" OR "einsalpha" OR "eldecalcitol" OR "elocalcitol" OR "endo d" OR "enstilar" OR "ercalcidiol" OR "ercalciol" OR "ergorone" OR "ergosterid" OR "ergosteride" OR "ergosterin activatum" OR "ergosterina irradiata" OR "ertron" OR "ertrone" OR "etalpha" OR "eurocal d3" OR "feroxyl" OR "feroxyle" OR "fortedol" OR "fortipan combi d" OR "fortodyl" OR "fortodyle" OR "fosamax plus d" OR "fosavance" OR "fultium-d3" OR "fultivit-d3" OR "genevis" OR "glicol d2" OR "hectorol" OR "hidroferol" OR "hitrol" OR "hydroxycalciferol" OR "hytakerol" OR "ideos" OR "idro steral" OR "idrosol d2" OR "inecalcitol" OR "infadin" OR "infadine" OR "infron" OR "infrone" OR "inovitan d" OR "ironco-b" OR "irradia" OR "irradian" OR "irradiated ergosterol" OR "isopropyl 1, 3, 24 trihydroxy 9, 10 secocholesta 5, 7, 10 (19), 22 tetraene 25 carboxylate" OR "issarlos" OR "kalciferol" OR "kh 1060" OR "kh1060" OR "kolkatriol" OR "kombi kalz" OR "kora liquid" OR "kosteo" OR "lemytriol" OR "leo 80185" OR "leo 90100" OR "leo 90105" OR "leo80185" OR "leo90100" OR "leo90105" OR "lexacalcitol" OR "lp 0113" OR "lp0113" OR "m 5181" OR "m5181" OR "manipal" OR "mastical d" OR "maxacalcitol" OR "maxi kalz vit d3" OR "maxi kalz vit. d3" OR "mc 1288" OR "mc 903" OR "mc2 01" OR "mc201" OR "mc903" OR "meditrol" OR "metadee" OR "mina d2" OR "mine d2" OR "mk 0217a" OR "mk0217a" OR "mulsiferol" OR "mykostin" OR "mykostine" OR "norsed combi d" OR "nycoplus calcigran" OR "oldevit" OR "oleovit d2" OR "one alpha" OR "onealfa" OR "one-alpha" OR "orocal d3" OR "orotre" OR "osseans d3" OR "ostelin" OR "osteline" OR "osteo d" OR "osteodina" OR "osteodine" OR "osteomerck" OR "osteotriol" OR "osteovit" OR "osteovitadin" OR "osteovitadine" OR "osteovitina" OR "osteovitine" OR "ostergil" OR "ostidil-d3" OR "ostoforte" OR "oxarol" OR "oxidevite" OR "oxydevit" OR "paracalcin" OR "paracalcitol" OR "paricalcitol" OR "parterol" OR "pefcalcitol" OR "plivit d" OR "poscal" OR "psorcutan" OR "psotriol" OR "qrx 101" OR "qrx101" OR "radiamon" OR "radiosterina" OR "radiosterine" OR "radiostol" OR "radsterin" OR "radsterine" OR "raquiferol d3" OR "raquiferol" OR "rayaldee" OR "renatriol" OR "rexamat" OR "ro 17 6218" OR "ro 21 5535" OR "ro 21 5816" OR "ro 215535" OR "ro 21-5816" OR "ro 23 4319" OR "ro 23 5112" OR "ro 23 5709" OR "ro 23 6005" OR "ro 23 6474" OR "ro 23 6710" OR "ro 23 7498" OR "ro 23 7553" OR "ro 23 7982" OR "ro 23 8525" OR "ro 23 9375" OR "ro 26 9228" OR "ro 26-9228" OR "ro 850" OR "ro21 5816" OR "ro215535" OR "ro21-5816" OR "ro26 9228" OR "ro26-9228" OR "rocaltrol" OR "roical" OR "rolsical" OR "sandocal-d" OR "seocalcitol" OR "shock ferol" OR "shockferol" OR "silkis" OR "sinervit d2" OR "sitriol" OR "soltriol" OR "sorilux" OR "steovit d3" OR "steovit forte" OR "steral" OR "steramin" OR "steramine" OR "sterobiol" OR "sterodin" OR "sterodine" OR "sterogyl 15" OR "sterogyl" OR "sterogyl-15" OR "sterosol" OR "sterovit" OR "sterovitina" OR "sterovitine" OR "tacal d3" OR "tacalcitol" OR "tachidon" OR "tachysterol, dihydro" OR "tachystin" OR "tachystine" OR "tachystol" OR "taclonex scalp" OR "taclonex" OR "tariol" OR "tepox cal d" OR "tetilan" OR "tevabone" OR "thorens" OR "tirocal" OR "tisocalcitate" OR "topitriol" OR "tri vit with fluoride" OR "triocalcit" OR "triple vita drops with fluoride" OR "tv 02" OR "u 32070" OR "u32070" OR "ucemine d" OR "ultranol" OR "unalfa" OR "un-alfa" OR "unalpha" OR "urto calciosterina" OR "urtosterina" OR "urtosterine" OR "uvedose" OR "uvesterol d" OR "valebo" OR "vantavo" OR "vectical" OR "versical d flas" OR "versical d" OR "vi de" OR "vi di" OR "vi-de 3" OR "vide" OR "videlta" OR "vidextra" OR "vidi" OR "vidiman" OR "vidolen" OR "vidue monico" OR "viduemonico" OR "vigantol" OR "vigoncal" OR "vigorsan" OR "vio d" OR "viosterin" OR "viosterine" OR "viosterol" OR "vitadit" OR "vitaplex" OR "vitasan d" OR "vitastabil d" OR "vitastabile d" OR "vitasterin" OR "vitasterine" OR "vitasterol" OR "vitavel d" OR "wandervit d2" OR "wynzora" OR "xamiol" OR "zemplar" OR "zk 156942" OR "zk156942" OR "lunacalcipol"):ti,ab,kw	
7	(#1 OR #2) AND (#3 OR #4 OR #5 OR #6) AND [embase]/lim	
8	#7 AND ("randomized controlled trial"/de or "controlled clinical study"/de or "randomization"/de or "intermethod comparison"/de or "double blind procedure"/de or "human experiment"/de OR (random* or placebo or "parallel group$" or crossover or "cross over" or assigned or allocated or volunteer or volunteers):ti,ab OR (open NEAR/1 label):ti,ab OR ((double or single or doubly or singly) NEAR/1 (blind or blinded or blindly)):ti,ab OR ((assign* or match or matched or allocation) NEAR/5 (alternate or group$ or intervention$ or patient$ or subject$ or participant$)):ti,ab OR (controlled NEAR/7 (study or design or trial)):ti,ab OR (compare or compared or comparison or trial):ti OR ((evaluated or evaluate or evaluating or assessed or assess) and (compare or compared or comparing or comparison)):ab) NOT (((random* NEAR/1 sampl* NEAR/7 ("cross section*" or questionnaire$ or survey* or database$)):ti,ab not ("comparative study"/de or "controlled study"/de or "randomi$ed controlled":ti,ab or "randomly assigned":ti,ab)) OR ("cross-sectional study"/de not ("randomized controlled trial"/de or "controlled clinical study"/de or "controlled study"/de or "randomi$ed controlled":ti,ab or "control	**125**

		group$":ti,ab)) OR ((((case NEAR/1 control*) and random*) not "randomi$ed controlled"):ti,ab) OR (("systematic review" not (trial or study)):ti) OR ((nonrandom* not random*):ti,ab) OR ("random field*":ti,ab) OR (("random cluster" NEAR/3 sampl*):ti,ab) OR ((review:ab and review/it) not trial:ti) OR ("we searched":ab and (review:ti or review/it)) OR ("update review":ab) OR ((databases NEAR/4 searched):ab) OR ((rat or rats or mouse or mice or swine or porcine or murine or sheep or lambs or pigs or piglets or rabbit or rabbits or cat or cats or dog or dogs or cattle or bovine or monkey or monkeys or trout or marmoset*):ti and "animal experiment"/de) OR ("animal experiment"/de not ("human experiment"/de or "human"/de)))<br><br>Filter Source: Box 3.e, Technical Supplement to Chapter 4: Searching for and Selecting Studies. Cochrane Handbook for Systematic Reviews of Interventions Version 6. (Syntax Translated from Ovid Embase to Elsevier Embase.com.)
**MEDLINE (Ovid)** and Epub Ahead of Print, In-Process, In-Data-Review & Other Non-Indexed Citations and Daily	1	exp "Dysmenorrhea"/ OR "Menstruation Disturbances"/ OR "Pelvic Pain"/ OR exp "Endometriosis"/
	2	(dysmenorrhea* OR dys-menorrhea* OR dysmenorrhoea* OR dys-menorrhoea* OR cramping OR endometrios* OR endometrioma* OR "adenomyos* externa" OR "e. externa" OR ((catamenial OR menstrua* OR menses OR premenstrua* OR period* OR pelvic OR pelvis) ADJ4 (pain* OR ache* OR cramp* OR distress*))).ti,ab,kf
	3	exp "Vitamin D"/
	4	((vitamin* ADJ4 D*) OR vitaminD* OR "vit D*" OR "vita D*" OR cholecalciferol* OR ergocalciferol*).ti,ab,kf
	5	(epivitamin* OR difvitamin* OR didehydrovitamin* OR dihydroxyvitamin* OR hydroxyvitamin* OR "25(OH)D" OR norvitamin* OR oxavitamin* OR oleovitamin* OR trihydroxyvitamin* OR epicholecalciferol* OR dihydroxycholecalciferol* OR didehydrocholecalciferol* OR hydroxycholecalciferol* OR norcholecalciferol* OR trihydroxycholecalciferol* OR epiergocalciferol* OR dihydroxyergocalciferol* OR hydroxyergocalciferol* OR trihydroxyergocalciferol* OR colecalciferol OR epicolecalciferol* OR dihydroxycolecalciferol* OR hydroxycolecalciferol* OR norcolecalciferol* OR oxacolecalciferol OR trihydroxycolecalciferol* OR dihydrotachysterol*).ti,ab,kf
	6	("1 [1alpha, 3beta dihydroxy 9, 10 secochola 5, 7, 10 (19) trien 24 oyl] piperidine" OR "1 fluoro 26, 27 dihomo 9, 10 secocholesta 5, 7, 10 (19), 16, 23 pentaene 3, 25 diol" OR "1, 3, 24 trihydroxy 9, 10 secocholesta 5, 7, 10 (19), 22 tetraene 25 carboxylic acid isopropyl ester" OR "19 nor 1, 25 dihydroxycalciferol" OR "19 nor 9, 10 seco 14beta cholesta 5, 7 dien 23 yne 1alpha, 3beta, 25 triol" OR "19 nor 9, 10 secoergosta 5, 7, 22 triene 1alpha, 3beta, 25 triol" OR "19 norcolecalciferol" OR "1alpha hydroxycalciferol" OR "1alpha, 25 dihydroxycalciferol" OR "1alpha-hydroxycalciferol" OR "1-alpha-hydroxycalciferol" OR "2 [(1, 3 dihydroxy 9, 10 secopregna 5, 7, 10 (19), 16 tetraen 20 yl) oxy] n (2, 2, 3, 3, 3 pentafluoropropyl) acetamide" OR "2 [(1alpha, 3beta dihydroxy 9, 10 secopregna 5, 7, 10 (19), 16 tetraen 20 yl) oxy] n (2, 2, 3, 3, 3 pentafluoropropyl) acetamide" OR "2 [1 [7 [2 (3, 5 dihydroxy 2 methylenecyclohexylidene) ethylidene] 3a, 4, 5, 6, 7, 7a hexahydro 3a methyl 1h inden 3 yl] ethoxy] n (2, 2, 3, 3, 3 pentafluoropropyl) acetamide" OR "20 [3 (1 hydroxy 1 methylethyl) benzyloxymethyl] 9, 10 secopregna 5, 7, 10 (19) triene 1alpha, 3beta diol" OR "20 [3 (2 hydroxypropan 2 yl) benzyloxymethyl] 9, 10 secopregna 5, 7, 10 (19) triene 1alpha, 3beta diol" OR "20 epi 22 ethoxy 24a,26a,27a trihomo 9,10 secocholesta 5,7,10(19) trien 23 yne 1alpha,3beta,25 triol" OR "20 epicalcitriol" OR "22 oxacalcitriol" OR "22, 23 dihydrocalciferol" OR "22,23 didehydrocalcitriol" OR "24 (2 methylpropane 2 sulfonyl) 9, 10 secochola 5, 7, 10 (19), 16, 23 pentaene 1, 3 diol" OR "24 cyclopropyl 9, 10 secochola 5, 7, 10 (19), 22 tetraene 1alpha, 3beta, 24 triol" OR "24 hydroxycalcidiol" OR "24 methyl 9, 10 secocholesta 5, 7, 10 (19), 22 tetraen 3 ol" OR "24, 25 dihydroxycalciferol" OR "24, 26, 27 trihomo 9, 10 secocholesta 5, 7, 10 (19), 22, 24 pentaene 1alpha, 3beta, 25 triol" OR "25 hydroxycalciferol" OR "26 hydroxycalcidiol" OR "26, 27 cyclo 9, 10 seco 5, 7, 10 (19), 22 cholestatetraene 1, 3, 24 triol" OR "2beta (3 hydroxypropoxy) 9, 10 secocholesta 5, 7, 10 (19) triene 1alpha, 3beta, 25 triol" OR "3 [2 [7a methyl 1 (6 methylheptan 2 yl) 2, 3, 3a, 5, 6, 7 hexahydro 1h inden 4 ylidene] ethylidene] 4 methylidenecyclohexan 1 ol" OR "4 [2 [1 (butan 2 yl) 7a methyloctahydro 4h inden 4 ylidene] ethylidene] 2 methylidenecyclohexane 1, 3 diol" OR "4 methylidene 5 [2 [7a methyl 1 [6 methylheptan 2 yl] 2, 3, 3a, 5, 6, 7 hexahydro 1h inden 4 ylidene] ethylidene] cyclohexane 1, 3 diol" OR "5 [2 [1 (5 hydroxy 1, 5 dimethylhexyl) 7a methyl 2, 3, 3a, 5, 6, 7 hexahydro 1h inden 4 ylidene] ethylidene] 4 methylenecyclohexane 1, 3 diol" OR "5 [2 [1 [5 cyclopropyl 5 hydroxypent 3 en 2 yl] 7a methyl 2, 3, 3a, 5, 6, 7 hexahydro 1h inden 4 ylidene] ethylidene] 4 methylidenecyclohexane 1, 3 diol" OR "5 [2 [1 [5, 6 dimethylhept 3 en 2 yl] 7a methyl 2, 3, 3a, 5, 6, 7 hexahydro 1h inden 4 ylidene] ethylidene] 4 methylidenecyclohexane 1, 3 diol" OR "5 [2 [1 [6 hydroxy 5, 6 dimethylhept 3 en 2 yl] 7a methyl 2, 3, 3a, 5, 6, 7 hexahydro 1h inden 4 ylidene] ethylidene] cyclohexane 1, 3 diol" OR "5 [2 [1 [6 hydroxy 6 methylheptan 2 yl] 7a methyl 2, 3, 3a, 5, 6, 7 hexahydro 1h inden 4 ylidene] ethylidene] 4 methylidenecyclohexane 1, 3 diol" OR "5 [2 [7a methyl 1 [6 methylheptan 2 yl] 2, 3, 3a, 5, 6, 7 hexahydro 1h inden 4 ylidene] ethylidene] 4 methylidenecyclohexane 1, 3 diol" OR "6 [4 [2 [5 hydroxy 2 methylidenecyclohexylidene] ethylidene] 7a methyl 2, 3, 3a, 5, 6, 7 hexahydro 1h inden 1 yl] 2 methyl 2, 3 heptanediol" OR "9, 10 seco 5, 7, 10 (19) cholestatrien 1alpha, 3beta

		diol" OR "9, 10 seco 5, 7, 10 (19) cholestatrien 3 ol" OR "9, 10 seco 5, 7, 10 (19) ergostatrien 3beta ol" OR "9, 10 seco 5, 7, 10 (19), 22 ergostatetraen 3beta ol" OR "9, 10 seco 5, 7, 10 (19), 22 ergostatetraene 1, 3 diol" OR "9, 10 seco 5, 7, 22 ergostatrien 3beta ol" OR "9, 10 secocholesta 5, 7, 10 (19) trien 3 ol" OR "9, 10 secocholesta 5, 7, 10 (19) triene 1alpha, 3beta diol" OR "9, 10 secocholesta 5, 7, 10 (19) triene 1alpha, 3beta, 25 triol" OR "9, 10 secocholesta 5, 7, 10 (19) triene 3beta, 24, 25 triol" OR "9, 10 secocholesta 5, 7, 10 (19) triene 3beta, 25 diol" OR "9, 10 secoergosta 5, 7, 10 (19) trien 3beta ol" OR "9, 10 secoergosta 5, 7, 10 (19), 22 tetraen 3 ol 19, 22 tetraen 3 ol" OR "9, 10 secoergosta 5, 7, 10, 22 tetraene 1, 3 diol" OR "9, 10 secoergosta 5, 7, 22 trien 3beta ol" OR "9,10 secocholesta 5,7,10(19) trien 23 yne 1,3,25 triol" OR "9,10 secocholesta 5,7,10(19) trien 23 yne 3,25 diol" OR "9,10 secocholesta 5,7,10(19),16 tetraen 23 yne 1,3,25 triol" OR "9,10 secocholesta 5,7,10(19),22 tetraene 1,3,25,26 tetrol" OR "a.t.10" OR "abt 358" OR "abt358" OR "activated 7 dehydrocholesterol" OR "adrovance" OR "afj d2" OR "alcovit d2" OR "aldevit" OR "alfacalcidol" OR "alfarol" OR "alpha calcidiol" OR "alpha calcidol" OR "alpha d3" OR "alphacalcidol" OR "antitanil" OR "antitetanin" OR "antitetanine" OR "arachitol" OR "asord" OR "at10" OR "atecen" OR "atocalcitol" OR "baby d" OR "becocalcidiol" OR "bentavit" OR "betamethasone dipropionate plus calcipotriene hydrate" OR "betamethasone dipropionate plus calcipotriene" OR "betamethasone dipropionate/calcipotriene hydrate" OR "betamethasone dipropionate/calcipotriene" OR "bocatriol" OR "bonalfa" OR "bonealpha" OR "bonesil d flas" OR "bonesyl" OR "bonky" OR "bxl 628" OR "bxl628" OR "cabone" OR "cacit d3" OR "cal d or" OR "cal d vita" OR "calcamin" OR "calcamine" OR "calceos" OR "calci chew d3 flex" OR "calci chew d3" OR "calcial d" OR "calcichew d3 extra" OR "calcichew d3 forte" OR "calcichew d3 opti" OR "calcichew d3" OR "calcidol" OR "calcifediol" OR "calciferol derivative" OR "calciferol" OR "calciferovit" OR "calcigran forte flex" OR "calcigran forte" OR "calcigran" OR "calcijex" OR "calcimagon d3 uno" OR "calcimagon d3" OR "calcimagon extra d3" OR "calcinosefaktor" OR "calcio d" OR "calciol" OR "calcioral d3" OR "calcipotriene hydrate plus betamethasone dipropionate" OR "calcipotriene hydrate" OR "calcipotriene hydrate/betamethasone dipropionate" OR "calcipotriene plus betamethasone dipropionate" OR "calcipotriene" OR "calcipotriene/betamethasone dipropionate" OR "calcipotriol" OR "calcitetrol" OR "calcitriol" OR "calcium d" OR "calcium wyeth" OR "calcivit d forte" OR "calcivit d" OR "caldefix" OR "calderol" OR "caldevita" OR "cal-d-or" OR "cal-d-vita" OR "calisvit" OR "calperos d3" OR "caltrate d" OR "caraben sc" OR "cb 1093" OR "cb1093" OR "chemovit d" OR "chocola d" OR "cicarol" OR "citrihexal" OR "citrokalciumd" OR "colextra-d3" OR "condol" OR "curatoderm" OR "d arthrin" OR "d arthrine" OR "d crivit" OR "d mulsin" OR "d tracetten" OR "d vatine" OR "d vital" OR "d2 vita" OR "d3 vicotrat" OR "dagravit d calcium" OR "daivobet" OR "daivonex" OR "dalonev" OR "davitamon d" OR "davitan" OR "davitin" OR "davonex" OR "ddrops" OR "decaps" OR "decostriol" OR "dediol" OR "dedrogyl" OR "dee osterol" OR "dee ron" OR "deeosterol" OR "deeron" OR "dekristol" OR "delakmin" OR "delta monovit" OR "deltabios" OR "deltalin" OR "deltaline" OR "deltamonovit" OR "deltar" OR "deltasterolo" OR "deltavit" OR "deltius" OR "deradion" OR "deradione" OR "deratol" OR "dergosten" OR "desunin" OR "desyn" OR "desyne" OR "detalup" OR "detamine" OR "deterapion" OR "deterapione" OR "devaron" OR "devitan" OR "devitil" OR "devitol" OR "dht intensol" OR "di actol" OR "di drol" OR "diactol" OR "dibase (drug)" OR "dibiovit" OR "dichistrolum" OR "dichysterol" OR "dichystrol" OR "didrogyl" OR "didrol" OR "didue vita" OR "diergin" OR "diergine" OR "diferol" OR "difilina" OR "difix" OR "dihydral" OR "dihydrotachysterin" OR "dihydrotachysterine" OR "dihydrotachysterol 2" OR "dihydrotachysterol 3" OR "dikystrol" OR "dilavit" OR "diserinal" OR "disir" OR "disnal" OR "disterina" OR "disterine" OR "ditelos" OR "divit urto" OR "divitina" OR "divitine" OR "diviturto" OR "divonex" OR "dn 101" OR "dn101" OR "dohyfral d" OR "dovobet" OR "dovonex" OR "doxercalciferol" OR "drisdol" OR "dumovit d" OR "duphafral" OR "dupharinterfran" OR "d-vital forte" OR "d-vital" OR "dydrogil" OR "dygratyl" OR "dz idrosol" OR "eb 1089" OR "eb1089" OR "ecalcidene" OR "ecatrol f" OR "ecatrol" OR "ed 71" OR "ed71" OR "einsalpha" OR "eldecalcitol" OR "elocalcitol" OR "endo d" OR "enstilar" OR "ercalcidiol" OR "ercalciol" OR "ergorone" OR "ergosterid" OR "ergosteride" OR "ergosterin activatum" OR "ergosterina irradiata" OR "ertron" OR "ertrone" OR "etalpha" OR "eurocal d3" OR "feroxyl" OR "feroxyle" OR "fortedol" OR "fortipan combi d" OR "fortodyl" OR "fortodyle" OR "fosamax plus d" OR "fosavance" OR "fultium-d3" OR "fultivit-d3" OR "genevis" OR "glicol d2" OR "hectorol" OR "hidroferol" OR "hitrol" OR "hydroxycalciferol" OR "hytakerol" OR "ideos" OR "idro steral" OR "idrosol d2" OR "inecalcitol" OR "infadin" OR "infadine" OR "infron" OR "infrone" OR "inovitan d" OR "ironco-b" OR "irradia" OR "irradian" OR "irradiated ergosterol" OR "isopropyl 1, 3, 24 trihydroxy 9, 10 secocholesta 5, 7, 10 (19), 22 tetraene 25 carboxylate" OR "issarlos" OR "kalciferol" OR "kh 1060" OR "kh1060" OR "kolkatriol" OR "kombi kalz" OR "kora liquid" OR "kosteo" OR "lemytriol" OR "leo 80185" OR "leo 90100" OR "leo 90105" OR "leo80185" OR "leo90100" OR "leo90105" OR "lexacalcitol" OR "lp 0113" OR "lp0113" OR "m 5181" OR "m5181" OR "manipal" OR "mastical d" OR "maxacalcitol" OR "maxi kalz vit d3" OR "maxi kalz vit. d3" OR "mc 1288" OR "mc 903" OR "mc2 01" OR "mc201" OR "mc903" OR "meditrol" OR "metadee" OR "mina d2" OR "mine d2" OR "mk 0217a" OR "mk0217a" OR "mulsiferol" OR "mykostin" OR "mykostine" OR "norsed combi d" OR "nycoplus calcigran" OR "oldevit" OR "oleovit d2" OR "one alpha" OR "onealfa" OR "one-alpha" OR "orocal d3" OR "orotre" OR "osseans d3" OR "ostelin" OR "osteline" OR "osteo d" OR "osteodina" OR "osteodine" OR "osteomerck" OR "osteotriol" OR "osteovit" OR "osteovitadin" OR "osteovitadine" OR "osteovitina" OR "osteovitine" OR "ostergil" OR "ostidil-d3" OR "ostoforte" OR "oxarol" OR "oxidevite" OR "oxydevit" OR "paracalcin" OR "paracalcitol" OR "paricalcitol" OR "parterol" OR "pefcalcitol" OR "plivit d" OR "poscal" OR "psorcutan" OR "psotriol" OR "qrx 101" OR "qrx101" OR "radiamon" OR "radiosterina" OR "radiosterine" OR "radiostol" OR "radsterin" OR "radsterine" OR "raquiferol d3" OR "raquiferol" OR "rayaldee" OR "renatriol" OR "rexamat" OR "ro 17 6218" OR "ro 21 5535" OR "ro 21 5816" OR "ro 215535" OR "ro 21-5816" OR "ro 23 4319" OR "ro 23 5112" OR "ro 23 5709" OR "ro 23 6005" OR "ro 23 6474" OR "ro 23 6710" OR "ro 23 7498" OR "ro 23 7553" OR "ro 23 7982" OR "ro 23 8525" OR "ro 23 9375" OR "ro 26 9228" OR "ro 26-9228" OR "ro 850" OR "ro21 5816" OR "ro215535" OR "ro21-5816" OR "ro26 9228" OR "ro26-9228" OR "rocaltrol" OR "roical" OR "rolsical" OR "sandocal-d" OR "seocalcitol" OR "shock ferol" OR "shockferol" OR "silkis" OR "sinervit d2" OR "sitriol" OR "soltriol" OR "sorilux" OR "steovit d3" OR "steovit forte" OR "steral" OR "steramin" OR "steramine" OR "sterobiol" OR "sterodin" OR "sterodine" OR "sterogyl 15" OR "sterogyl" OR "sterogyl-15" OR "sterosol" OR "sterovit" OR "sterovitina" OR "sterovitine" OR "tacal d3" OR "tacalcitol" OR "tachidon" OR "tachysterol, dihydro" OR "tachystin" OR "tachystine" OR "tachystol" OR "taclonex scalp" OR "taclonex" OR "tariol" OR "tepox cal d" OR "tetilan" OR "tevabone" OR "thorens" OR "tirocal" OR "tisocalcitate" OR "topitriol" OR "tri vit with fluoride" OR "triocalcit" OR "triple vita drops with fluoride" OR "tv 02" OR "u 32070" OR "u32070" OR "ucemine d" OR "ultranol" OR "unalfa" OR "un-alfa" OR "unalpha" OR "urto calciosterina" OR "urtosterina" OR "urtosterine" OR "uvedose" OR "uvesterol d" OR "valebo" OR "vantavo" OR "vectical" OR "versical d flas" OR "versical d" OR "vi de" OR "vi di" OR "vi-de 3" OR "vide" OR "videlta" OR "vidextra" OR "vidi" OR "vidiman" OR "vidolen" OR "vidue monico" OR "viduemonico" OR "vigantol" OR "vigoncal" OR "vigorsan" OR "vio d" OR "viosterin" OR "viosterine" OR "viosterol" OR "vitadit" OR "vitaplex" OR "vitasan d" OR "vitastabil d" OR "vitastabile d" OR "vitasterin" OR "vitasterine" OR "vitasterol" OR "vitavel d" OR "wandervit d2" OR "wynzora" OR "xamiol" OR "zemplar" OR "zk 156942" OR "zk156942" OR "lunacalcipol").ti,ab,kf	
	7	(1 OR 2) AND (3 OR 4 OR 5 OR 6)	
	8	7 AND (randomized controlled trial.pt. or controlled clinical trial.pt. or randomi*ed.ab. or placebo.ab. or drug therapy.fs. or randomly.ab. or trial.ab. or groups.ab. not (exp animals/ not humans.sh.))<br><br>Filter Source: Box 3.c, Technical Supplement to Chapter 4: Searching for and Selecting Studies. Cochrane Handbook for Systematic Reviews of Interventions Version 6. (Add: randomised.ab)	**157**
**Cochrane Library**	1	[mh "Dysmenorrhea"] OR [mh ^"Menstruation Disturbances"] OR [mh ^"Pelvic Pain"] OR [mh "Endometriosis"]	
including	2	(dysmenorrhea* OR dys-menorrhea* OR dysmenorrhoea* OR dys-menorrhoea* OR cramping OR endometrios* OR endometrioma* OR	

registers from ICTRP and ClinicalTrials.gov		(adenomyos* NEXT/1 externa) OR "e. externa" OR ((catamenial OR menstrua* OR menses OR premenstrua* OR period* OR pelvic OR pelvis) NEAR/3 (pain* OR ache* OR cramp* OR distress*))):ti,ab,kw	
	3	[mh "Vitamin D"]	
	4	(((vitamin* OR vita OR vit) NEAR/3 (D or D1 or D2 or D3 or D4 or D5 or D6 or D7)) OR vitaminD* OR cholecalciferol* OR ergocalciferol*):ti,ab,kw	
	5	(epivitamin* OR difvitamin* OR didehydrovitamin* OR dihydroxyvitamin* OR hydroxyvitamin* OR "25(OH)D" OR norvitamin* OR oxavitamin* OR oleovitamin* OR trihydroxyvitamin* OR epicholecalciferol* OR dihydroxycholecalciferol* OR didehydrocholecalciferol* OR hydroxycholecalciferol* OR norcholecalciferol* OR trihydroxycholecalciferol* OR epiergocalciferol* OR dihydroxyergocalciferol* OR hydroxyergocalciferol* OR trihydroxyergocalciferol* OR colecalciferol OR epicolecalciferol* OR dihydroxycolecalciferol* OR hydroxycolecalciferol* OR norcolecalciferol* OR oxacolecalciferol OR trihydroxycolecalciferol* OR dihydrotachysterol*):ti,ab,kw	
	6	(#1 OR #2) AND (#3 OR #4 OR #5)	
	7	#6 Limits in Cochrane Reviews and Trials	**170**
**1) CINAHL Plus with Full Text (EBSCOhost)**	1	mh ("Dysmenorrhea+" OR "Menstruation Disorders" OR "Pelvic Pain" OR "Endometriosis+")	
	2	dysmenorrhea* OR dys-menorrhea* OR dysmenorrhoea* OR dys-menorrhoea* OR cramping OR endometrios* OR endometrioma* OR "adenomyos* externa" OR "e. externa" OR ((catamenial OR menstrua* OR menses OR premenstrua* OR period* OR pelvic OR pelvis) N3 (pain* OR ache* OR cramp* OR distress*))	
	3	mh ("Vitamin D+")	
	4	(vitamin* N3 D*) OR vitaminD* OR "vit D*" OR "vita D*" OR cholecalciferol* OR ergocalciferol*	
	5	epivitamin* OR difvitamin* OR didehydrovitamin* OR dihydroxyvitamin* OR hydroxyvitamin* OR "25(OH)D" OR norvitamin* OR oxavitamin* OR oleovitamin* OR trihydroxyvitamin* OR epicholecalciferol* OR dihydroxycholecalciferol* OR didehydrocholecalciferol* OR hydroxycholecalciferol* OR norcholecalciferol* OR trihydroxycholecalciferol* OR epiergocalciferol* OR dihydroxyergocalciferol* OR hydroxyergocalciferol* OR trihydroxyergocalciferol* OR colecalciferol OR epicolecalciferol* OR dihydroxycolecalciferol* OR hydroxycolecalciferol* OR norcolecalciferol* OR oxacolecalciferol OR trihydroxycolecalciferol* OR dihydrotachysterol*	

6	"1 [1alpha, 3beta dihydroxy 9, 10 secochola 5, 7, 10 (19) trien 24 oyl] piperidine" OR "1 fluoro 26, 27 dihomo 9, 10 secocholesta 5, 7, 10 (19), 16, 23 pentaene 3, 25 diol" OR "1, 3, 24 trihydroxy 9, 10 secocholesta 5, 7, 10 (19), 22 tetraene 25 carboxylic acid isopropyl ester" OR "19 nor 1, 25 dihydroxycalciferol" OR "19 nor 9, 10 seco 14beta cholesta 5, 7 dien 23 yne 1alpha, 3beta, 25 triol" OR "19 nor 9, 10 secoergosta 5, 7, 22 triene 1alpha, 3beta, 25 triol" OR "19 norcolecalciferol" OR "1alpha hydroxycalciferol" OR "1alpha, 25 dihydroxycalciferol" OR "1alpha-hydroxycalciferol" OR "1-alpha-hydroxycalciferol" OR "2 [(1, 3 dihydroxy 9, 10 secopregna 5, 7, 10 (19), 16 tetraen 20 yl) oxy] n (2, 2, 3, 3, 3 pentafluoropropyl) acetamide" OR "2 [(1alpha, 3beta dihydroxy 9, 10 secopregna 5, 7, 10 (19), 16 tetraen 20 yl) oxy] n (2, 2, 3, 3, 3 pentafluoropropyl) acetamide" OR "2 [1 [7 [2 (3, 5 dihydroxy 2 methylenecyclohexylidene) ethylidene] 3a, 4, 5, 6, 7, 7a hexahydro 3a methyl 1h inden 3 yl] ethoxy] n (2, 2, 3, 3, 3 pentafluoropropyl) acetamide" OR "20 [3 (1 hydroxy 1 methylethyl) benzyloxymethyl] 9, 10 secopregna 5, 7, 10 (19) triene 1alpha, 3beta diol" OR "20 [3 (2 hydroxypropan 2 yl) benzyloxymethyl] 9, 10 secopregna 5, 7, 10 (19) triene 1alpha, 3beta diol" OR "20 epi 22 ethoxy 24a,26a,27a trihomo 9,10 secocholesta 5,7,10(19) trien 23 yne 1alpha,3beta,25 triol" OR "20 epicalcitriol" OR "22 oxacalcitriol" OR "22, 23 dihydrocalciferol" OR "22,23 didehydrocalcitriol" OR "24 (2 methylpropane 2 sulfonyl) 9, 10 secochola 5, 7, 10 (19), 16, 23 pentaene 1, 3 diol" OR "24 cyclopropyl 9, 10 secochola 5, 7, 10 (19), 22 tetraene 1alpha, 3beta, 24 triol" OR "24 hydroxycalcidiol" OR "24 methyl 9, 10 secocholesta 5, 7, 10 (19), 22 tetraen 3 ol" OR "24, 25 dihydroxycalciferol" OR "24, 26, 27 trihomo 9, 10 secocholesta 5, 7, 10 (19), 22, 24 pentaene 1alpha, 3beta, 25 triol" OR "25 hydroxycalciferol" OR "26 hydroxycalcidiol" OR "26, 27 cyclo 9, 10 seco 5, 7, 10 (19), 22 cholestatetraene 1, 3, 24 triol" OR "2beta (3 hydroxypropoxy) 9, 10 secocholesta 5, 7, 10 (19) triene 1alpha, 3beta, 25 triol" OR "3 [2 [7a methyl 1 (6 methylheptan 2 yl) 2, 3, 3a, 5, 6, 7 hexahydro 1h inden 4 ylidene] ethylidene] 4 methylidenecyclohexan 1 ol" OR "4 [2 [1 (butan 2 yl) 7a methyloctahydro 4h inden 4 ylidene] ethylidene] 2 methylidenecyclohexane 1, 3 diol" OR "4 methylidene 5 [2 [7a methyl 1 [6 methylheptan 2 yl] 2, 3, 3a, 5, 6, 7 hexahydro 1h inden 4 ylidene] ethylidene] cyclohexane 1, 3 diol" OR "5 [2 [1 (5 hydroxy 1, 5 dimethylhexyl) 7a methyl 2, 3, 3a, 5, 6, 7 hexahydro 1h inden 4 ylidene] ethylidene] 4 methylenecyclohexane 1, 3 diol" OR "5 [2 [1 [5 cyclopropyl 5 hydroxypent 3 en 2 yl] 7a methyl 2, 3, 3a, 5, 6, 7 hexahydro 1h inden 4 ylidene] ethylidene] 4 methylidenecyclohexane 1, 3 diol" OR "5 [2 [1 [5, 6 dimethylhept 3 en 2 yl] 7a methyl 2, 3, 3a, 5, 6, 7 hexahydro 1h inden 4 ylidene] ethylidene] 4 methylidenecyclohexane 1, 3 diol" OR "5 [2 [1 [6 hydroxy 5, 6 dimethylhept 3 en 2 yl] 7a methyl 2, 3, 3a, 5, 6, 7 hexahydro 1h inden 4 ylidene] ethylidene] cyclohexane 1, 3 diol" OR "5 [2 [1 [6 hydroxy 6 methylheptan 2 yl] 7a methyl 2, 3, 3a, 5, 6, 7 hexahydro 1h inden 4 ylidene] ethylidene] 4 methylidenecyclohexane 1, 3 diol" OR "5 [2 [7a methyl 1 [6 methylheptan 2 yl] 2, 3, 3a, 5, 6, 7 hexahydro 1h inden 4 ylidene] ethylidene] 4 methylidenecyclohexane 1, 3 diol" OR "6 [4 [2 [5 hydroxy 2 methylidenecyclohexylidene] ethylidene] 7a methyl 2, 3, 3a, 5, 6, 7 hexahydro 1h inden 1 yl] 2 methyl 2, 3 heptanediol" OR "9, 10 seco 5, 7, 10 (19) cholestatrien 1alpha, 3beta diol" OR "9, 10 seco 5, 7, 10 (19) cholestatrien 3 ol" OR "9, 10 seco 5, 7, 10 (19) ergostatrien 3beta ol" OR "9, 10 seco 5, 7, 10 (19), 22 ergostatetraen 3beta ol" OR "9, 10 seco 5, 7, 10 (19), 22 ergostatetraene 1, 3 diol" OR "9, 10 seco 5, 7, 22 ergostatrien 3beta ol" OR "9, 10 secocholesta 5, 7, 10 (19) trien 3 ol" OR "9, 10 secocholesta 5, 7, 10 (19) triene 1alpha, 3beta diol" OR "9, 10 secocholesta 5, 7, 10 (19) triene 1alpha, 3beta, 25 triol" OR "9, 10 secocholesta 5, 7, 10 (19) triene 3beta, 24, 25 triol" OR "9, 10 secocholesta 5, 7, 10 (19) triene 3beta, 25 diol" OR "9, 10 secoergosta 5, 7, 10 (19) trien 3beta ol" OR "9, 10 secoergosta 5, 7, 10 (19), 22 tetraen 3 ol 19, 22 tetraen 3 ol" OR "9, 10 secoergosta 5, 7, 10, 22 tetraene 1, 3 diol" OR "9, 10 secoergosta 5, 7, 22 trien 3beta ol" OR "9,10 secocholesta 5,7,10(19) trien 23 yne 1,3,25 triol" OR "9,10 secocholesta 5,7,10(19) trien 23 yne 3,25 diol" OR "9,10 secocholesta 5,7,10(19),16 tetraen 23 yne 1,3,25 triol" OR "9,10 secocholesta 5,7,10(19),22 tetraene 1,3,25,26 tetrol" OR "a.t.10" OR "abt 358" OR "abt358" OR "activated 7 dehydrocholesterol" OR "adrovance" OR "afj d2" OR "alcovit d2" OR "aldevit" OR "alfacalcidol" OR "alfarol" OR "alpha calcidiol" OR "alpha calcidol" OR "alpha d3" OR "alphacalcidol" OR "antitanil" OR "antitetanin" OR "antitetanine" OR "arachitol" OR "asord" OR "at10" OR "at-10" OR "atecen" OR "atocalcitol" OR "baby d" OR "becocalcidiol" OR "bentavit" OR "betamethasone dipropionate plus calcipotriene hydrate" OR "betamethasone dipropionate plus calcipotriene" OR "betamethasone dipropionate/calcipotriene hydrate" OR "betamethasone dipropionate/calcipotriene" OR "bocatriol" OR "bonalfa" OR "bonealpha" OR "bonesil d flas" OR "bonesyl" OR "bonky" OR "bxl 628" OR "bxl628" OR "cabone" OR "cacit d3" OR "cal d or" OR "cal d vita" OR "calcamin" OR "calcamine" OR "calceos" OR "calci chew d3 flex" OR "calci chew d3" OR "calcial d" OR "calcichew d3 extra" OR "calcichew d3 forte" OR "calcichew d3 opti" OR "calcichew d3" OR "calcidol" OR "calcifediol" OR "calciferol derivative" OR "calciferol" OR "calciferovit" OR "calcigran forte flex" OR "calcigran forte" OR "calcigran" OR "calcijex" OR "calcimagon d3 uno" OR "calcimagon d3" OR "calcimagon extra d3" OR "calcinosefaktor" OR "calcio d" OR "calciol" OR "calcioral d3" OR "calcipotriene hydrate plus betamethasone dipropionate" OR "calcipotriene hydrate" OR "calcipotriene hydrate/betamethasone dipropionate" OR "calcipotriene plus betamethasone dipropionate" OR "calcipotriene" OR "calcipotriene/betamethasone dipropionate" OR "calcipotriol" OR "calcitetrol" OR "calcitriol" OR "calcium d" OR "calcium wyeth" OR "calcivit d forte" OR "calcivit d" OR "caldefix" OR "calderol" OR "caldevita" OR "cal-d-or" OR "cal-d-vita" OR "calisvit" OR "calperos d3" OR "caltrate d" OR "caraben sc" OR "cb 1093" OR "cb1093" OR "chemovit d" OR "chocola d" OR "cicarol" OR "citrihexal" OR "citrokalciumd" OR "colextra-d3" OR "condol" OR "curatoderm" OR "d arthrin" OR "d arthrine" OR "d crivit" OR "d mulsin" OR "d tracetten" OR "d vatine" OR "d vital" OR "d2 vita" OR "d3 vicotrat" OR "dagravit d calcium" OR "daivobet" OR "daivonex" OR "dalonev" OR "davitamon d" OR "davitan" OR "davitin" OR "davonex" OR "ddrops" OR "decaps" OR "decostriol" OR "dediol" OR "dedrogyl" OR "dee osterol" OR "dee ron" OR "deeosterol" OR "deeron" OR "dekristol" OR "delakmin" OR "delta monovit" OR "deltabios" OR "deltalin" OR "deltaline" OR "deltamonovit" OR "deltar" OR "deltasterolo" OR "deltavit" OR "deltius" OR "deradion" OR "deradione" OR "deratol" OR "dergosten" OR "desunin" OR "desyn" OR "desyne" OR "detalup" OR "detamine" OR "deterapion" OR "deterapione" OR "devaron" OR "devitan" OR "devitil" OR "devitol" OR "dht intensol" OR "di actol" OR "di drol" OR "diactol" OR "dibase (drug)" OR "dibiovit" OR "dichistrolum" OR "dichysterol" OR "dichystrol" OR "didrogyl" OR "didrol" OR "didue vita" OR "diergin" OR "diergine" OR "diferol" OR "difilina" OR "difix" OR "dihydral" OR "dihydrotachysterin" OR "dihydrotachysterine" OR "dihydrotachysterol 2" OR "dihydrotachysterol 3" OR "dikystrol" OR "dilavit" OR "diserinal" OR "disir" OR "disnal" OR "disterina" OR "disterine" OR "ditelos" OR "divit urto" OR "divitina" OR "divitine" OR "diviturto" OR "divonex" OR "dn 101" OR "dn101" OR "dohyfral d" OR "dovobet" OR "dovonex" OR "doxercalciferol" OR "drisdol" OR "dumovit d" OR "duphafral" OR "dupharinterfran" OR "d-vital forte" OR "d-vital" OR "dydrogil" OR "dygratyl" OR "dz idrosol" OR "eb 1089" OR "eb1089" OR "ecalcidene" OR "ecatrol f" OR "ecatrol" OR "ed 71" OR "ed71" OR "einsalpha" OR "eldecalcitol" OR "elocalcitol" OR "endo d" OR "enstilar" OR "ercalcidiol" OR "ercalciol" OR "ergorone" OR "ergosterid" OR "ergosteride" OR "ergosterin activatum" OR "ergosterina irradiata" OR "ertron" OR "ertrone" OR "etalpha" OR "eurocal d3" OR "feroxyl" OR "feroxyle" OR "fortedol" OR "fortipan combi d" OR "fortodyl" OR "fortodyle" OR "fosamax plus d" OR "fosavance" OR "fultium-d3" OR "fultivit-d3" OR "genevis" OR "glicol d2" OR "hectorol" OR "hidroferol" OR "hitrol" OR "hydroxycalciferol" OR "hytakerol" OR "ideos" OR "idro steral" OR "idrosol d2" OR "inecalcitol" OR "infadin" OR "infadine" OR "infron" OR "infrone" OR "inovitan d" OR "ironco-b" OR "irradia" OR "irradian" OR "irradiated ergosterol" OR "isopropyl 1, 3, 24 trihydroxy 9, 10 secocholesta 5, 7, 10 (19), 22 tetraene 25 carboxylate" OR "issarlos" OR "kalciferol" OR "kh 1060" OR "kh1060" OR "kolkatriol" OR "kombi kalz" OR "kora liquid" OR "kosteo" OR "lemytriol" OR "leo 80185" OR "leo 90100" OR "leo 90105" OR "leo80185" OR "leo90100" OR "leo90105" OR "lexacalcitol" OR "lp 0113" OR "lp0113" OR "m 5181" OR "m5181" OR "manipal" OR "mastical d" OR "maxacalcitol" OR "maxi kalz vit d3" OR "maxi kalz vit. d3" OR "mc 1288" OR "mc 903" OR "mc2 01" OR "mc201" OR "mc903" OR "meditrol" OR "metadee" OR "mina d2" OR "mine d2" OR "mk 0217a" OR "mk0217a" OR "mulsiferol" OR "mykostin" OR "mykostine" OR "norsed combi d" OR "nycoplus calcigran" OR "oldevit" OR "oleovit d2" OR "one alpha" OR "onealfa" OR "one-alpha" OR "orocal d3" OR "orotre" OR "osseans d3" OR "ostelin" OR "osteline" OR "osteo d" OR "osteodina" OR "osteodine" OR "osteomerck" OR "osteotriol" OR "osteovit" OR "osteovitadin" OR "osteovitadine" OR "osteovitina" OR "osteovitine" OR "ostergil" OR "ostidil-d3" OR "ostoforte" OR "oxarol" OR "oxidevite" OR "oxydevit" OR "paracalcin" OR "paracalcitol" OR "paricalcitol" OR "parterol" OR "pefcalcitol" OR "plivit d" OR "poscal" OR "psorcutan" OR "psotriol" OR "qrx 101" OR "qrx101" OR "radiamon" OR "radiosterina" OR "radiosterine" OR "radiostol" OR "radsterin" OR "radsterine" OR "raquiferol d3" OR "raquiferol" OR "rayaldee" OR "renatriol" OR "rexamat" OR "ro 17 6218" OR "ro 21 5535" OR "ro 21 5816" OR "ro 215535" OR "ro 21-5816" OR "ro 23 4319" OR "ro 23 5112" OR "ro 23 5709" OR "ro 23 6005" OR "ro 23 6474" OR "ro 23 6710" OR "ro 23 7498" OR "ro 23 7553" OR "ro 23 7982" OR "ro 23 8525" OR "ro 23 9375" OR "ro 26 9228" OR "ro 26-9228" OR "ro 850" OR "ro21 5816" OR "ro215535" OR "ro21-5816" OR "ro26 9228" OR "ro26-9228" OR "rocaltrol" OR "roical" OR "rolsical" OR "sandocal-d" OR "seocalcitol" OR "shock ferol" OR "shockferol" OR "silkis" OR "sinervit d2" OR "sitriol" OR "soltriol" OR "sorilux" OR "steovit d3" OR "steovit forte" OR "steral" OR "steramin" OR "steramine" OR "sterobiol" OR "sterodin" OR "sterodine" OR "sterogyl 15" OR "sterogyl" OR "sterogyl-15" OR "sterosol" OR "sterovit" OR "sterovitina" OR "sterovitine" OR "tacal d3" OR "tacalcitol" OR "tachidon" OR "tachysterol, dihydro" OR "tachystin" OR "tachystine" OR "tachystol" OR "taclonex scalp" OR "taclonex" OR "tariol" OR "tepox cal d" OR "tetilan" OR "tevabone" OR "thorens" OR "tirocal" OR "tisocalcitate" OR "topitriol" OR "tri vit with fluoride" OR "triocalcit" OR "triple vita drops with fluoride" OR "tv 02" OR "u 32070" OR "u32070" OR "ucemine d" OR "ultranol" OR "unalfa" OR "un-alfa" OR "unalpha" OR "urto calciosterina" OR "urtosterina" OR "urtosterine" OR "uvedose" OR "uvesterol d" OR "valebo" OR "vantavo" OR "vectical" OR "versical d flas" OR "versical d" OR "vi de" OR "vi di" OR "vi-de 3" OR "vide" OR "videlta" OR "vidextra" OR "vidi" OR "vidiman" OR "vidolen" OR "vidue monico" OR "viduemonico" OR "vigantol" OR "vigoncal" OR "vigorsan" OR "vio d" OR "viosterin" OR "viosterine" OR "viosterol" OR "vitadit" OR "vitaplex" OR "vitasan d" OR "vitastabil d" OR "vitastabile d" OR "vitasterin" OR "vitasterine" OR "vitasterol" OR "vitavel d" OR "wandervit d2" OR "wynzora" OR "xamiol" OR "zemplar" OR "zk 156942" OR "zk156942" OR "lunacalcipol"

	7	(s1 OR s2) AND (s3 OR s4 OR s5 OR s6)	
	8	s7 AND ((MH ("randomized controlled trials" OR "double-blind studies" OR "single-blind studies" OR "random assignment" OR "pretest-posttest design" OR "cluster sample") OR TI (randomised OR randomized) OR AB (random*) OR TI (trial) OR (MH (sample size) AND AB (assigned OR allocated OR control)) OR MH (placebos) OR PT (randomized controlled trial) OR AB (control W5 group) OR MH ("crossover design" OR "comparative studies") OR AB (cluster W3 RCT)) NOT ((MH ("animals+" OR "animal studies") OR TI (animal model*)) NOT MH (human)))<br><br>Filter Source: Box 3.f., Technical Supplement to Chapter 4: Searching for and Selecting Studies. Cochrane Handbook for Systematic Reviews of Interventions Version 6.	**32**
**2)**<br>**Scopus**	1	title-abs (dysmenorrhea* OR dys-menorrhea* OR dysmenorrhoea* OR dys-menorrhoea* OR cramping OR endometrios* OR endometrioma* OR "adenomyos* externa" OR "e. externa" OR ((catamenial OR menstrua* OR menses OR premenstrua* OR period* OR pelvic OR pelvis) W/3 (pain* OR ache* OR cramp* OR distress*))) OR authkey (dysmenorrhea* OR dys-menorrhea* OR dysmenorrhoea* OR dys-menorrhoea* OR cramping OR endometrios* OR endometrioma* OR "adenomyos* externa" OR "e. externa" OR ((catamenial OR menstrua* OR menses OR premenstrua* OR period* OR pelvic OR pelvis) W/3 (pain* OR ache* OR cramp* OR distress*)))	
	2	title-abs ((vitamin* W/3 D*) OR vitaminD* OR "vit D*" OR "vita D*" OR cholecalciferol* OR ergocalciferol*) OR authkey ((vitamin* W/3 D*) OR vitaminD* OR "vit D*" OR "vita D*" OR cholecalciferol* OR ergocalciferol*)	
	3	title-abs (epivitamin* OR difvitamin* OR didehydrovitamin* OR dihydroxyvitamin* OR hydroxyvitamin* OR "25(OH)D" OR norvitamin* OR oxavitamin* OR oleovitamin* OR trihydroxyvitamin* OR epicholecalciferol* OR dihydroxycholecalciferol* OR didehydrocholecalciferol* OR hydroxycholecalciferol* OR norcholecalciferol* OR trihydroxycholecalciferol* OR epiergocalciferol* OR dihydroxyergocalciferol* OR hydroxyergocalciferol* OR trihydroxyergocalciferol* OR colecalciferol OR epicolecalciferol* OR dihydroxycolecalciferol* OR hydroxycolecalciferol* OR norcolecalciferol* OR oxacolecalciferol OR trihydroxycolecalciferol* OR dihydrotachysterol*) OR authkey (epivitamin* OR difvitamin* OR didehydrovitamin* OR dihydroxyvitamin* OR hydroxyvitamin* OR "25(OH)D" OR norvitamin* OR oxavitamin* OR oleovitamin* OR trihydroxyvitamin* OR epicholecalciferol* OR dihydroxycholecalciferol* OR didehydrocholecalciferol* OR hydroxycholecalciferol* OR norcholecalciferol* OR trihydroxycholecalciferol* OR epiergocalciferol* OR dihydroxyergocalciferol* OR hydroxyergocalciferol* OR trihydroxyergocalciferol* OR colecalciferol OR epicolecalciferol* OR dihydroxycolecalciferol* OR hydroxycolecalciferol* OR norcolecalciferol* OR oxacolecalciferol OR	

	trihydroxycolecalciferol* OR dihydrotachysterol*)
4	title-abs ("at10" OR "atecen" OR "atocalcitol" OR "baby d" OR "becocalcidiol" OR "bentavit" OR "betamethasone dipropionate plus calcipotriene hydrate" OR "betamethasone dipropionate plus calcipotriene" OR "betamethasone dipropionate/calcipotriene hydrate" OR "betamethasone dipropionate/calcipotriene" OR "bocatriol" OR "bonalfa" OR "bonealpha" OR "bonesil d flas" OR "bonesyl" OR "bonky" OR "bxl 628" OR "bxl628" OR "cabone" OR "cacit d3" OR "cal d or" OR "cal d vita" OR "calcamin" OR "calcamine" OR "calceos" OR "calci chew d3 flex" OR "calci chew d3" OR "calcial d" OR "calcichew d3 extra" OR "calcichew d3 forte" OR "calcichew d3 opti" OR "calcichew d3" OR "calcidol" OR "calcifediol" OR "calciferol derivative" OR "calciferol" OR "calciferovit" OR "calcigran forte flex" OR "calcigran forte" OR "calcigran" OR "calcijex" OR "calcimagon d3 uno" OR "calcimagon d3" OR "calcimagon extra d3" OR "calcinosefaktor" OR "calcio d" OR "calciol" OR "calcioral d3" OR "calcipotriene hydrate plus betamethasone dipropionate" OR "calcipotriene hydrate" OR "calcipotriene hydrate/betamethasone dipropionate" OR "calcipotriene plus betamethasone dipropionate" OR "calcipotriene" OR "calcipotriene/betamethasone dipropionate" OR "calcipotriol" OR "calcitetrol" OR "calcitriol" OR "calcium d" OR "calcium wyeth" OR "calcivit d forte" OR "calcivit d" OR "caldefix" OR "calderol" OR "caldevita" OR "cal-d-or" OR "cal-d-vita" OR "calisvit" OR "calperos d3" OR "caltrate d" OR "caraben sc" OR "cb 1093" OR "cb1093" OR "chemovit d" OR "chocola d" OR "cicarol" OR "citrihexal" OR "citrokalciumd" OR "colextra-d3" OR "condol" OR "curatoderm" OR "d arthrin" OR "d arthrine" OR "d crivit" OR "d mulsin" OR "d tracetten" OR "d vatine" OR "d vital" OR "d2 vita" OR "d3 vicotrat" OR "dagravit d calcium" OR "daivobet" OR "daivonex" OR "dalonev" OR "davitamon d" OR "davitan" OR "davitin" OR "davonex" OR "ddrops" OR "decaps" OR "decostriol" OR "dediol" OR "dedrogyl" OR "dee osterol" OR "dee ron" OR "deeosterol" OR "deeron" OR "dekristol" OR "delakmin" OR "delta monovit" OR "deltabios" OR "deltalin" OR "deltaline" OR "deltamonovit" OR "deltar" OR "deltasterolo" OR "deltavit" OR "deltius" OR "deradion" OR "deradione" OR "deratol" OR "dergosten" OR "desunin" OR "desyn" OR "desyne" OR "detalup" OR "detamine" OR "deterapion" OR "deterapione" OR "devaron" OR "devitan" OR "devitil" OR "devitol" OR "dht intensol" OR "di actol" OR "di drol" OR "diactol" OR "dibase (drug)" OR "dibiovit" OR "dichistrolum" OR "dichysterol" OR "dichystrol" OR "didrogyl" OR "didrol" OR "didue vita" OR "diergin" OR "diergine" OR "diferol" OR "difilina" OR "difix" OR "dihydral" OR "dihydrotachysterin" OR "dihydrotachysterine" OR "dihydrotachysterol 2" OR "dihydrotachysterol 3" OR "dikystrol" OR "dilavit" OR "diserinal" OR "disir" OR "disnal" OR "disterina" OR "disterine" OR "ditelos" OR "divit urto" OR "divitina" OR "divitine" OR "diviturto" OR "divonex" OR "dn 101" OR "dn101" OR "dohyfral d" OR "dovobet" OR "dovonex" OR "doxercalciferol" OR "drisdol" OR "dumovit d" OR "duphafral" OR "dupharinterfran" OR "d-vital forte" OR "d-vital" OR "dydrogil" OR "dygratyl" OR "dz idrosol" OR "eb 1089" OR "eb1089" OR "ecalcidene" OR "ecatrol f" OR "ecatrol" OR "ed 71" OR "ed71" OR "einsalpha" OR "eldecalcitol" OR "elocalcitol" OR "endo d" OR "enstilar" OR "ercalcidiol" OR "ercalciol" OR "ergorone" OR "ergosterid" OR "ergosteride" OR "ergosterin activatum" OR "ergosterina irradiata" OR "ertron" OR "ertrone" OR "etalpha" OR "eurocal d3" OR "feroxyl" OR "feroxyle" OR "fortedol" OR "fortipan combi d" OR "fortodyl" OR "fortodyle" OR "fosamax plus d" OR "fosavance" OR "fultium-d3" OR "fultivit-d3" OR "genevis" OR "glicol d2" OR "hectorol" OR "hidroferol" OR "hitrol" OR "hydroxycalciferol" OR "hytakerol" OR "ideos" OR "idro steral" OR "idrosol d2" OR "inecalcitol" OR "infadin" OR "infadine" OR "infron" OR "infrone" OR "inovitan d" OR "ironco-b" OR "irradia" OR "irradian" OR "irradiated ergosterol" OR "issarlos" OR "kalciferol" OR "kh 1060" OR "kh1060" OR "kolkatriol" OR "kombi kalz" OR "kora liquid" OR "kosteo" OR "lemytriol" OR "leo 80185" OR "leo 90100" OR "leo 90105" OR "leo80185" OR "leo90100" OR "leo90105" OR "lexacalcitol" OR "lp 0113" OR "lp0113" OR "m 5181" OR "m5181" OR "manipal" OR "mastical d" OR "maxacalcitol" OR "maxi kalz vit d3" OR "maxi kalz vit. d3" OR "mc 1288" OR "mc 903" OR "mc2 01" OR "mc201" OR "mc903" OR "meditrol" OR "metadee" OR "mina d2" OR "mine d2" OR "mk 0217a" OR "mk0217a" OR "mulsiferol" OR "mykostin" OR "mykostine" OR "norsed combi d" OR "nycoplus calcigran" OR "oldevit" OR "oleovit d2" OR "one alpha" OR "onealfa" OR "one-alpha" OR "orocal d3" OR "orotre" OR "osseans d3" OR "ostelin" OR "osteline" OR "osteo d" OR "osteodina" OR "osteodine" OR "osteomerck" OR "osteotriol" OR "osteovit" OR "osteovitadin" OR "osteovitadine" OR "osteovitina" OR "osteovitine" OR "ostergil" OR "ostidil-d3" OR "ostoforte" OR "oxarol" OR "oxidevite" OR "oxydevit" OR "paracalcin" OR "paracalcitol" OR "paricalcitol" OR "parterol" OR "pefcalcitol" OR "plivit d" OR "poscal" OR "psorcutan" OR "psotriol" OR "qrx 101" OR "qrx101" OR "radiamon" OR "radiosterina" OR "radiosterine" OR "radiostol" OR "radsterin" OR "radsterine" OR "raquiferol d3" OR "raquiferol" OR "rayaldee" OR "renatriol" OR "rexamat" OR "ro 17 6218" OR "ro 21 5535" OR "ro 21 5816" OR "ro 215535" OR "ro 21-5816" OR "ro 23 4319" OR "ro 23 5112" OR "ro 23 5709" OR "ro 23 6005" OR "ro 23 6474" OR "ro 23 6710" OR "ro 23 7498" OR "ro 23 7553" OR "ro 23 7982" OR "ro 23 8525" OR "ro 23 9375" OR "ro 26 9228" OR "ro 26-9228" OR "ro 850" OR "ro21 5816" OR "ro215535" OR "ro21-5816" OR "ro26 9228" OR "ro26-9228" OR "rocaltrol" OR "roical" OR "rolsical" OR "sandocal-d" OR "seocalcitol" OR "shock ferol" OR "shockferol" OR "silkis" OR "sinervit d2" OR "sitriol" OR "soltriol" OR "sorilux" OR "steovit d3" OR "steovit forte" OR "steral" OR "steramin" OR "steramine" OR "sterobiol" OR "sterodin" OR "sterodine" OR "sterogyl 15" OR "sterogyl" OR "sterogyl-15" OR "sterosol" OR "sterovit" OR "sterovitina" OR "sterovitine" OR "tacal d3" OR "tacalcitol" OR "tachidon" OR "tachysterol, dihydro" OR "tachystin" OR "tachystine" OR "tachystol" OR "taclonex scalp" OR "taclonex" OR "tariol" OR "tepox cal d" OR "tetilan" OR "tevabone" OR "thorens" OR "tirocal" OR "tisocalcitate" OR "topitriol" OR "tri vit with fluoride" OR "triocalcit" OR "triple vita drops with fluoride" OR "tv 02" OR "u 32070" OR "u32070" OR "ucemine d" OR "ultranol" OR "unalfa" OR "un-alfa" OR "unalpha" OR "urto calciosterina" OR "urtosterina" OR "urtosterine" OR "uvedose" OR "uvesterol d" OR "valebo" OR "vantavo" OR "vectical" OR "versical d flas" OR "versical d" OR "vi de" OR "vi di" OR "vi-de 3" OR "vide" OR "videlta" OR "vidextra" OR "vidi" OR "vidiman" OR "vidolen" OR "vidue monico" OR "viduemonico" OR "vigantol" OR "vigoncal" OR "vigorsan" OR "vio d" OR "viosterin" OR "viosterine" OR "viosterol" OR "vitadit" OR "vitaplex" OR "vitasan d" OR "vitastabil d" OR "vitastabile d" OR "vitasterin" OR "vitasterine" OR "vitasterol" OR "vitavel d" OR "wandervit d2" OR "wynzora" OR "xamiol" OR "zemplar" OR "zk 156942" OR "zk156942" OR "lunacalcipol") OR authkey ("at10" OR "atecen" OR "atocalcitol" OR "baby d" OR "becocalcidiol" OR "bentavit" OR "betamethasone dipropionate plus calcipotriene hydrate" OR "betamethasone dipropionate plus calcipotriene" OR "betamethasone dipropionate/calcipotriene hydrate" OR "betamethasone dipropionate/calcipotriene" OR "bocatriol" OR "bonalfa" OR "bonealpha" OR "bonesil d flas" OR "bonesyl" OR "bonky" OR "bxl 628" OR "bxl628" OR "cabone" OR "cacit d3" OR "cal d or" OR "cal d vita" OR "calcamin" OR "calcamine" OR "calceos" OR "calci chew d3 flex" OR "calci chew d3" OR "calcial d" OR "calcichew d3 extra" OR "calcichew d3 forte" OR "calcichew d3 opti" OR "calcichew d3" OR "calcidol" OR "calcifediol" OR "calciferol derivative" OR "calciferol" OR "calciferovit" OR "calcigran forte flex" OR "calcigran forte" OR "calcigran" OR "calcijex" OR "calcimagon d3 uno" OR "calcimagon d3" OR "calcimagon extra d3" OR "calcinosefaktor" OR "calcio d" OR "calciol" OR "calcioral d3" OR "calcipotriene hydrate plus betamethasone dipropionate" OR "calcipotriene hydrate" OR "calcipotriene hydrate/betamethasone dipropionate" OR "calcipotriene plus betamethasone dipropionate" OR "calcipotriene" OR "calcipotriene/betamethasone dipropionate" OR "calcipotriol" OR "calcitetrol" OR "calcitriol" OR "calcium d" OR "calcium wyeth" OR "calcivit d forte" OR "calcivit d" OR "caldefix" OR "calderol" OR "caldevita" OR "cal-d-or" OR "cal-d-vita" OR "calisvit" OR "calperos d3" OR "caltrate d" OR "caraben sc" OR "cb 1093" OR "cb1093" OR "chemovit d" OR "chocola d" OR "cicarol" OR "citrihexal" OR "citrokalciumd" OR "colextra-d3" OR "condol" OR "curatoderm" OR "d arthrin" OR "d arthrine" OR "d crivit" OR "d mulsin" OR "d tracetten" OR "d vatine" OR "d vital" OR "d2 vita" OR "d3 vicotrat" OR "dagravit d calcium" OR "daivobet" OR "daivonex" OR "dalonev" OR "davitamon d" OR "davitan" OR "davitin" OR "davonex" OR "ddrops" OR "decaps" OR "decostriol" OR "dediol" OR "dedrogyl" OR "dee osterol" OR "dee ron" OR "deeosterol" OR "deeron" OR "dekristol" OR "delakmin" OR "delta monovit" OR "deltabios" OR "deltalin" OR "deltaline" OR "deltamonovit" OR "deltar" OR "deltasterolo" OR "deltavit" OR "deltius" OR "deradion" OR "deradione" OR "deratol" OR "dergosten" OR "desunin" OR "desyn" OR "desyne" OR "detalup" OR "detamine" OR "deterapion" OR "deterapione" OR "devaron" OR "devitan" OR "devitil" OR "devitol" OR "dht intensol" OR "di actol" OR "di drol" OR "diactol" OR "dibase (drug)" OR "dibiovit" OR "dichistrolum" OR "dichysterol" OR "dichystrol" OR "didrogyl" OR "didrol" OR "didue vita" OR "diergin" OR "diergine" OR "diferol" OR "difilina" OR "difix" OR "dihydral" OR "dihydrotachysterin" OR "dihydrotachysterine" OR "dihydrotachysterol 2" OR "dihydrotachysterol 3" OR "dikystrol" OR "dilavit" OR "diserinal" OR "disir" OR "disnal" OR "disterina" OR "disterine" OR "ditelos" OR "divit urto" OR "divitina" OR "divitine" OR "diviturto" OR "divonex" OR "dn 101" OR "dn101" OR "dohyfral d" OR "dovobet" OR "dovonex" OR "doxercalciferol" OR "drisdol" OR "dumovit d" OR "duphafral" OR "dupharinterfran" OR "d-vital forte" OR "d-vital" OR "dydrogil" OR "dygratyl" OR "dz idrosol" OR "eb 1089" OR "eb1089" OR "ecalcidene" OR "ecatrol f" OR "ecatrol" OR "ed 71" OR "ed71" OR "einsalpha" OR "eldecalcitol" OR "elocalcitol" OR "endo d" OR "enstilar" OR "ercalcidiol" OR "ercalciol" OR "ergorone" OR "ergosterid" OR "ergosteride" OR "ergosterin activatum" OR "ergosterina irradiata" OR "ertron" OR "ertrone" OR "etalpha" OR "eurocal d3" OR "feroxyl" OR "feroxyle" OR "fortedol" OR "fortipan combi d" OR "fortodyl" OR "fortodyle" OR "fosamax plus d" OR "fosavance" OR "fultium-d3" OR "fultivit-d3" OR "genevis" OR "glicol d2" OR "hectorol" OR "hidroferol" OR "hitrol" OR "hydroxycalciferol" OR "hytakerol" OR "ideos" OR "idro steral" OR "idrosol d2" OR "inecalcitol" OR "infadin" OR "infadine" OR "infron" OR "infrone" OR "inovitan d" OR "ironco-b" OR "irradia" OR "irradian" OR "irradiated ergosterol" OR "issarlos" OR "kalciferol" OR "kh 1060" OR "kh1060" OR "kolkatriol" OR "kombi kalz" OR "kora liquid" OR "kosteo" OR "lemytriol" OR "leo 80185" OR "leo 90100" OR "leo 90105" OR "leo80185" OR "leo90100" OR "leo90105" OR "lexacalcitol" OR "lp 0113" OR "lp0113" OR "m 5181" OR "m5181" OR "manipal" OR "mastical d" OR "maxacalcitol" OR "maxi kalz vit d3" OR "maxi kalz vit. d3" OR "mc 1288" OR "mc 903" OR "mc2 01" OR "mc201" OR "mc903" OR "meditrol" OR "metadee" OR "mina d2" OR "mine d2" OR "mk 0217a" OR "mk0217a" OR "mulsiferol" OR "mykostin" OR "mykostine" OR "norsed combi d" OR "nycoplus calcigran" OR "oldevit" OR "oleovit d2" OR "one alpha" OR "onealfa" OR "one-alpha" OR "orocal d3" OR "orotre" OR "osseans d3" OR "ostelin" OR

	"osteline" OR "osteo d" OR "osteodina" OR "osteodine" OR "osteomerck" OR "osteotriol" OR "osteovit" OR "osteovitadin" OR "osteovitadine" OR "osteovitina" OR "osteovitine" OR "ostergil" OR "ostidil-d3" OR "ostoforte" OR "oxarol" OR "oxidevite" OR "oxydevit" OR "paracalcin" OR "paracalcitol" OR "paricalcitol" OR "parterol" OR "pefcalcitol" OR "plivit d" OR "poscal" OR "psorcutan" OR "psotriol" OR "qrx 101" OR "qrx101" OR "radiamon" OR "radiosterina" OR "radiosterine" OR "radiostol" OR "radsterin" OR "radsterine" OR "raquiferol d3" OR "raquiferol" OR "rayaldee" OR "renatriol" OR "rexamat" OR "ro 17 6218" OR "ro 21 5535" OR "ro 21 5816" OR "ro 215535" OR "ro 21-5816" OR "ro 23 4319" OR "ro 23 5112" OR "ro 23 5709" OR "ro 23 6005" OR "ro 23 6474" OR "ro 23 6710" OR "ro 23 7498" OR "ro 23 7553" OR "ro 23 7982" OR "ro 23 8525" OR "ro 23 9375" OR "ro 26 9228" OR "ro 26-9228" OR "ro 850" OR "ro21 5816" OR "ro215535" OR "ro21-5816" OR "ro26 9228" OR "ro26-9228" OR "rocaltrol" OR "roical" OR "rolsical" OR "sandocal-d" OR "seocalcitol" OR "shock ferol" OR "shockferol" OR "silkis" OR "sinervit d2" OR "sitriol" OR "soltriol" OR "sorilux" OR "steovit d3" OR "steovit forte" OR "steral" OR "steramin" OR "steramine" OR "sterobiol" OR "sterodin" OR "sterodine" OR "sterogyl 15" OR "sterogyl" OR "sterogyl-15" OR "sterosol" OR "sterovit" OR "sterovitina" OR "sterovitine" OR "tacal d3" OR "tacalcitol" OR "tachidon" OR "tachysterol, dihydro" OR "tachystin" OR "tachystine" OR "tachystol" OR "taclonex scalp" OR "taclonex" OR "tariol" OR "tepox cal d" OR "tetilan" OR "tevabone" OR "thorens" OR "tirocal" OR "tisocalcitate" OR "topitriol" OR "tri vit with fluoride" OR "triocalcit" OR "triple vita drops with fluoride" OR "tv 02" OR "u 32070" OR "u32070" OR "ucemine d" OR "ultranol" OR "unalfa" OR "un-alfa" OR "unalpha" OR "urto calciosterina" OR "urtosterina" OR "urtosterine" OR "uvedose" OR "uvesterol d" OR "valebo" OR "vantavo" OR "vectical" OR "versical d flas" OR "versical d" OR "vi de" OR "vi di" OR "vi-de 3" OR "vide" OR "videlta" OR "vidextra" OR "vidi" OR "vidiman" OR "vidolen" OR "vidue monico" OR "viduemonico" OR "vigantol" OR "vigoncal" OR "vigorsan" OR "vio d" OR "viosterin" OR "viosterine" OR "viosterol" OR "vitadit" OR "vitaplex" OR "vitasan d" OR "vitastabil d" OR "vitastabile d" OR "vitasterin" OR "vitasterine" OR "vitasterol" OR "vitavel d" OR "wandervit d2" OR "wynzora" OR "xamiol" OR "zemplar" OR "zk 156942" OR "zk156942" OR "lunacalcipol")	
5	(INDEXTERMS ("clinical trials" OR "clinical trials as a topic" OR "randomized controlled trial" OR "Randomized Controlled Trials as Topic" OR "controlled clinical trial" OR "Controlled Clinical Trials" OR "random allocation" OR "Double-Blind Method" OR "Single-Blind Method" OR "Cross-Over Studies" OR "Placebos" OR "multicenter study" OR "double blind procedure" OR "single blind procedure" OR "crossover procedure" OR "clinical trial" OR "controlled study" OR "randomization" OR "placebo")) OR (TITLE-ABS-KEY (("clinical trials" OR "clinical trials as a topic" OR "randomized controlled trial" OR "Randomized Controlled Trials as Topic" OR "controlled clinical trial" OR "Controlled Clinical Trials as Topic" OR "random allocation" OR "randomly allocated" OR "allocated randomly" OR "Double-Blind Method" OR "Single-Blind Method" OR "Cross-Over Studies" OR "Placebos" OR "cross-over trial" OR "single blind" OR "double blind" OR "factorial design" OR "factorial trial"))) OR (TITLE (clinical trial OR trial OR rct* OR random* OR blind*))<br><br>Filter Source: This filter is built by NUS Medical Library using MESH and EMTREE terms as Index terms and Keywords. Useful to search in Scopus for RCTs.	
6	#1 AND (#2 OR #3 OR #4) AND #5	**166**

Supplementary Table S2. Sensitivity analyses for the primary outcome

**Sensitivity analysis**	**Study N**	**Pooled MD (95%CI)**	***p*-value**	**I^2(%)**
Using fixed effect model	11	-1.26 (-1.50 to -1.02)	<0.001	82.05
Low risk of bias	6	-1.53 (-2.12 to -0.95)	<0.001	26.88
Double blinded studies	8	-1.59 (-2.18 to -1.00)	<0.001	50.93
Non-funded study	2	-1.84 (-2.87 to -0.81)	<0.001	76.30
Definite placebo use	8	-1.59 (-2.18 to -1.00)	<0.001	50.93

CI, confidence interval; MD mean difference

Supplementary Figure S1. Quality assessment of the included studies using the RoB-2 tool

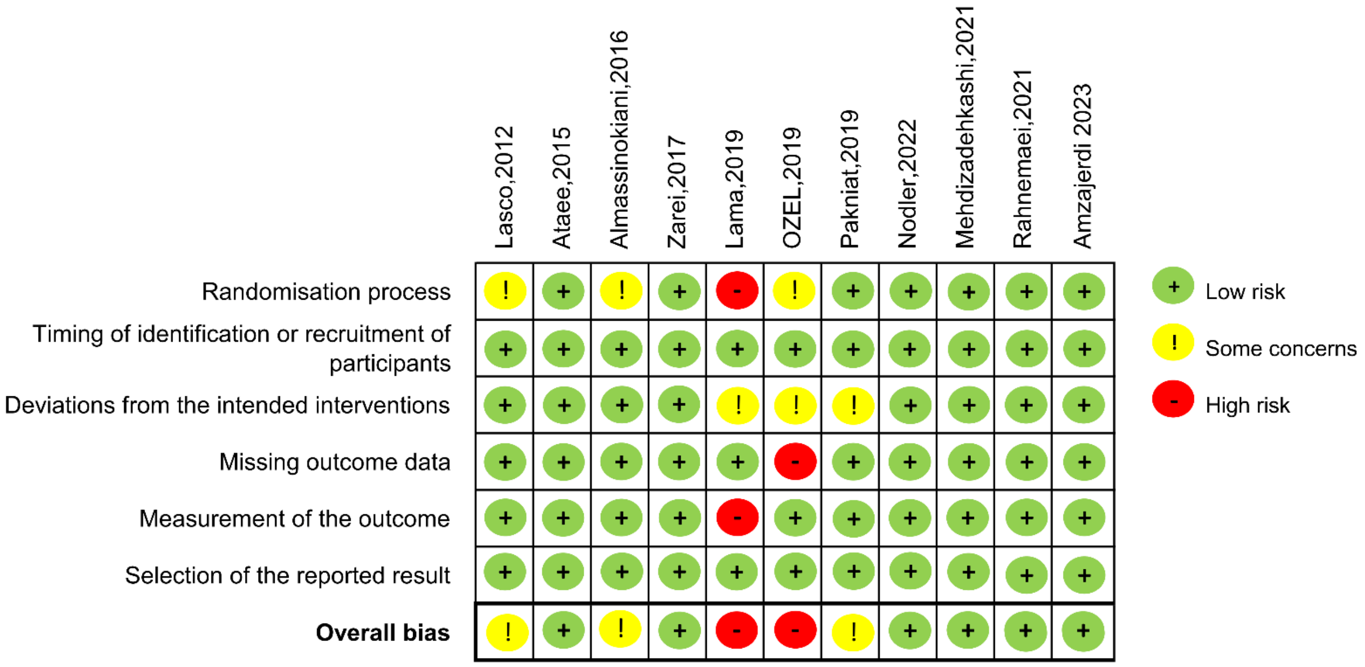

Supplementary Figure S2. Funnel plot

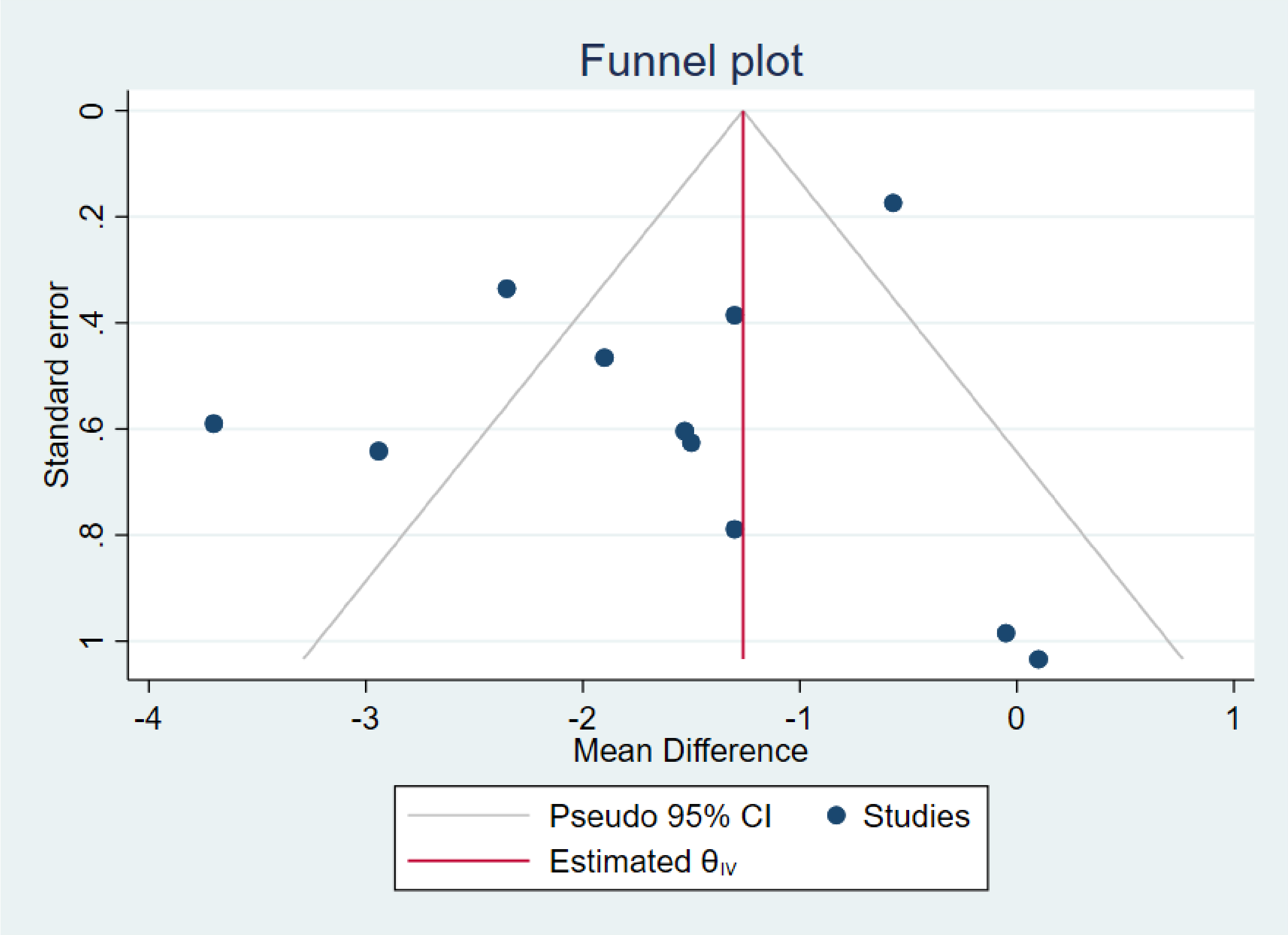

Supplementary Figure S3. Leave-one-out analysis

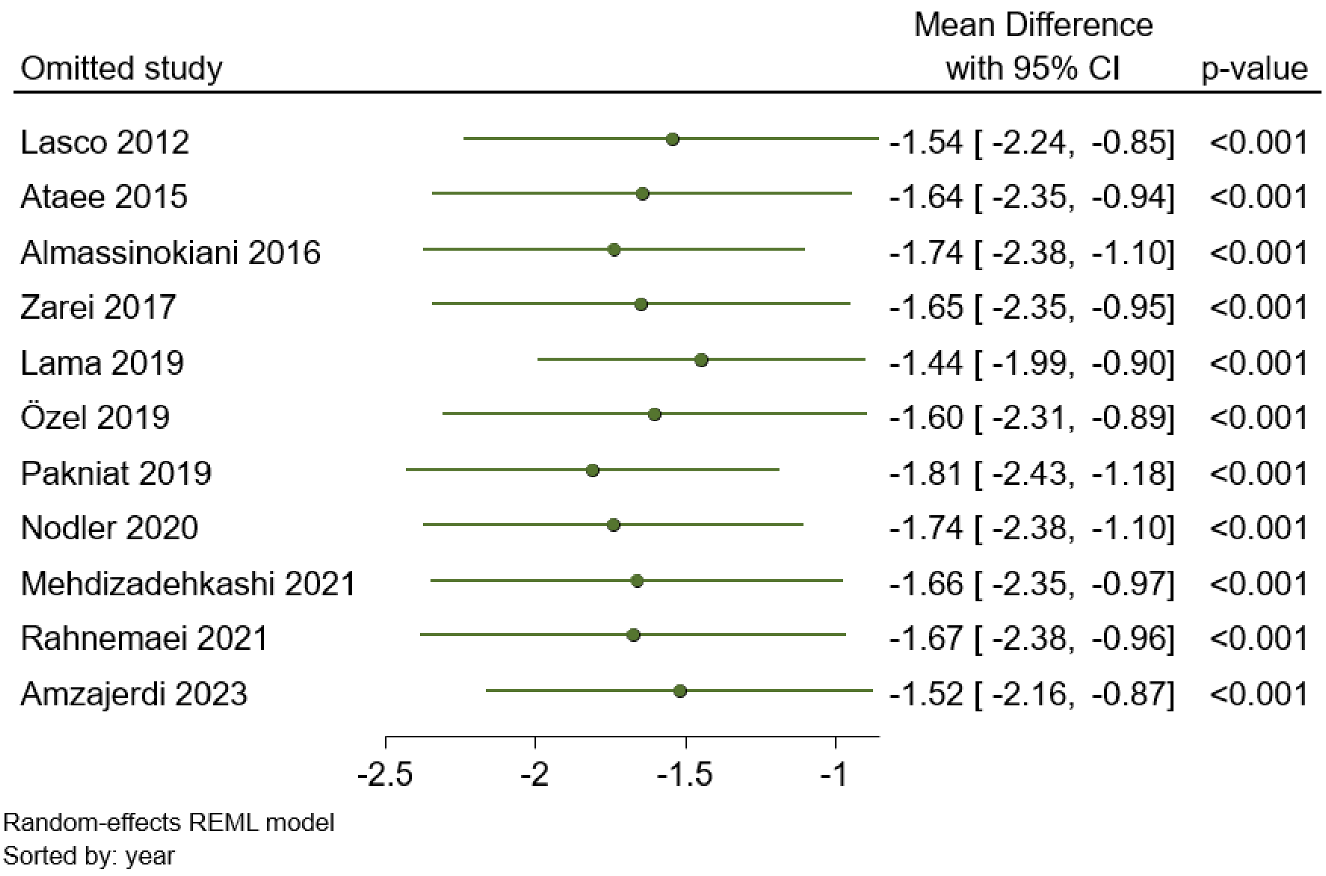

Sensitivity analysis was conducted by removing one trial at a time to determine what influence each study had on the pooled analysis. The pooled result seemed to be robust. For example, removing the study conducted by Özel et al in 2019 only changed the pooled estimate from -1.64 to -160 (95% CI -2.31 to -0.89; $p < 0.001$). CI, confidence interval; REML, restricted maximum likelihood

Supplementary Figure S4. The use of rescue analgesics among patients with dysmenorrhoea

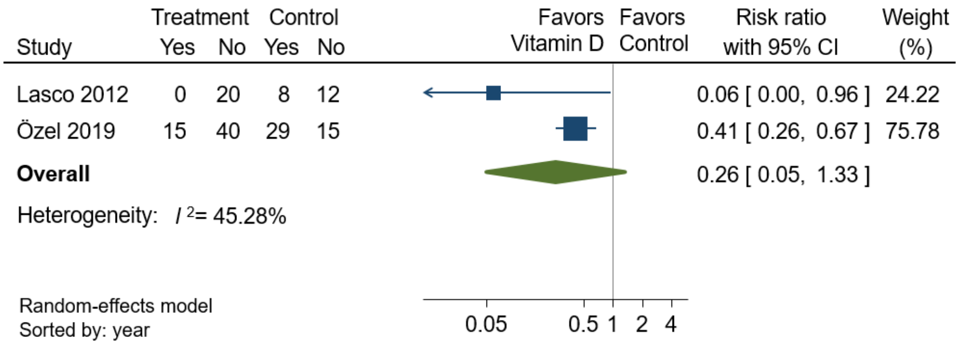

CI, confidence interval; RR, rate ratio
